# Fitting power-laws in empirical data with estimators that work for all exponents

**DOI:** 10.1371/journal.pone.0170920

**Published:** 2017-02-28

**Authors:** Rudolf Hanel, Bernat Corominas-Murtra, Bo Liu, Stefan Thurner

**Affiliations:** 1 Section for Science of Complex Systems, Medical University of Vienna, Spitalgasse 23, 1090 Vienna, Austria; 2 Santa Fe Institute, 1399 Hyde Park Road, Santa Fe, NM 87501, United States of America; 3 IIASA, Schlossplatz 1, 2361 Laxenburg, Austria; 4 Complexity Science Hub Vienna, Josefstädterstrasse 39, A-1090 Vienna, Austria; University of Sydney, AUSTRALIA

## Abstract

Most standard methods based on maximum likelihood (ML) estimates of power-law exponents can only be reliably used to identify exponents smaller than minus one. The argument that power laws are otherwise not normalizable, depends on the underlying sample space the data is drawn from, and is true only for sample spaces that are unbounded from above. Power-laws obtained from bounded sample spaces (as is the case for practically all data related problems) are always free of such limitations and maximum likelihood estimates can be obtained for arbitrary powers without restrictions. Here we first derive the appropriate ML estimator for arbitrary exponents of power-law distributions on bounded discrete sample spaces. We then show that an almost identical estimator also works perfectly for continuous data. We implemented this ML estimator and discuss its performance with previous attempts. We present a general recipe of how to use these estimators and present the associated computer codes.

## Introduction

The omnipresence of power-laws in natural, socio-economic, technical, and living systems has triggered immense research activity to understand their origins. It has become clear in the past decades that there exist several distinct ways to generate power-laws (or asymptotic power-laws), for an overview see for example [1, 2]. In short, power-laws of the form

$$p\left(x\right)=Cx^{-\lambda }\;,$$ (1)

arise in critical phenomena [3, 4], in systems displaying self-organized criticality [5], preferential attachment type of processes [6–9], multiplicative processes with constraints [10], systems described by generalized entropies [11, 12], or sample space reducing processes [13], i.e. processes that reduce the number of possible outcomes (sample space) as they unfold. Literally thousands of physical, natural, man-made, social, and cultural processes exhibit power-laws, the most famous being earthquake magnitudes [14, 15], city sizes [16, 17], foraging and distribution pattern of various animal species [18], evolutionary extinction events [19], or the frequency of word occurrences in languages, known as Zipf’s law [20].

It is obvious that estimating power-law exponents from data is a task that sometimes should be done with high precision. For example if one wants to determine the universality class a given process belongs to, or when one estimates probabilities of extreme events. In such situations small errors in the estimation of exponents may lead to dramatically wrong predictions with potentially serious consequences.

Estimating power-law exponents from data is not an entirely trivial task. Many reported power-laws are simply not exact power-laws, but follow other distribution functions. Such partial or imperfect power-laws come in various flavours: power-laws with an exponential cut-off, asymptotic power-laws, *q*-exponential distributions, which approach exact power-laws only in the tail of the distribution function. Other families of distribution functions may possess one or more regions where a power-law fit may be reasonable. Strategies for identifying such regions in imperfect power-law distributions may depend on the scenario. In some scenarios, e.g. for *q*-exponential distribution functions, ML algorithms that are specifically tailored to fit the particular class of distribution functions, may be considered. When no parametric representation of an imperfect power-law distribution is available, other strategies may be followed. For instance, screening the data for ranges where the null-hypothesis, that the data has been sampled from an exact power-law, can not be rejected for a particular significance level. Despite the importance of developing adequate methods for distinguishing real power-laws from alternative hypotheses, we will, except for some remarks, not address this issue in depth here, since good standard literature on the topic of Bayesian *alternative hypotheses testing* exists, see for example [21, 22]. For power-laws some of these matters have been discussed also in [23]. In particular, the possibility to use the Kolmogorov-Smirnov (KS) goodness of fit test (GOF) [24, 25] for finding optimal fitting ranges. In which sense the KS GOF-test is adequate in this context and how so called p-values of the KS GOF-test needs to be interpreted is a non-trivial question. The p-value of the KS GOF-test does not directly correspond to the rate of falsely rejected power-laws. The two-sided KS GOF-test in fact rejects an unacceptably large percentage of samples drawn from exact power-laws. What the two-sided KS GOF-test in fact rejects or accepts at a certain confidence level, is that the power-law with the estimated exponent represent the same distribution function as the data, which has been used to estimate the exponent. For more details on how to calibrate critical values of the statistical parameter used in the KS GOF-test in order to control the false rejection rate of power-laws with respect to the estimator, see S3 File appendix C.

Here we simply focus on estimating power-law exponents from data on a sound probabilistic basis, using a classic Bayesian parameter estimation approach, see e.g. [26, 27], that provides us with *maximum likelihood* (ML) estimators for estimating power-law exponents over the full range of reasonably accessible values. Other approaches restrict the limit of application to power-laws with exponents smaller than −1 [23, 28, 29]. Having such estimators without any a priori restriction of the exponent value is of particular interest for a large classes of situations where exponents close to λ ∼ 1 appear (Zipf’s law). We will argue here that whenever dealing with data we can assume discrete and bounded samples spaces (domains), which guarantees that power-laws are normalizable for arbitrary powers λ. We then show that the corresponding ML estimator can then also be used to estimate exponents from data that is sampled from continuous sample spaces, or from sample spaces that are not bounded from above. Our approach is equivalent to the one provided in [27].

### Questions before fitting power-laws

In physics the theoretical understanding of a process sometimes provides us with the luxury of knowing the exact form of the distribution function that one has to fit to the data. For instance think of critical phenomena such as Ising magnets in 2 dimensions at the critical temperature, where it is understood that the susceptibility follows a power-law of the form (*T* − *T*_*c*_)^−*γ*^, with *γ* a critical exponent, that occasionally even can be predicted mathematically. However, often—and especially when dealing with complex systems—we do not enjoy this luxury and usually do not know the exact functions to fit to the data.

In such a case, let us imagine that you have a data set and from first inspection you think that a power-law fit could be a reasonable thing to do. It is then essential, before starting with the fitting procedures, to clarify what one knows about the process that generated this data. The following questions may help to do so.

- Do you have information about the dynamics of the process that is generating what appears to be a power-law?
- Is the data generated by a Bernoulli process (e.g. tossing dice), or not (e.g. preferential attachment)?
- Is the data available as a collection of samples (a list of measurements), or only coarse-grained in form of a histogram (binned or aggregated data).
- Is the data sampled from a discrete (e.g. text) or continuous sample space (e.g. earthquakes)?
- Does the data have a natural ordering (e.g. magnitudes of earthquakes), or not (e.g. word frequencies in texts)?

**Fig 1:**
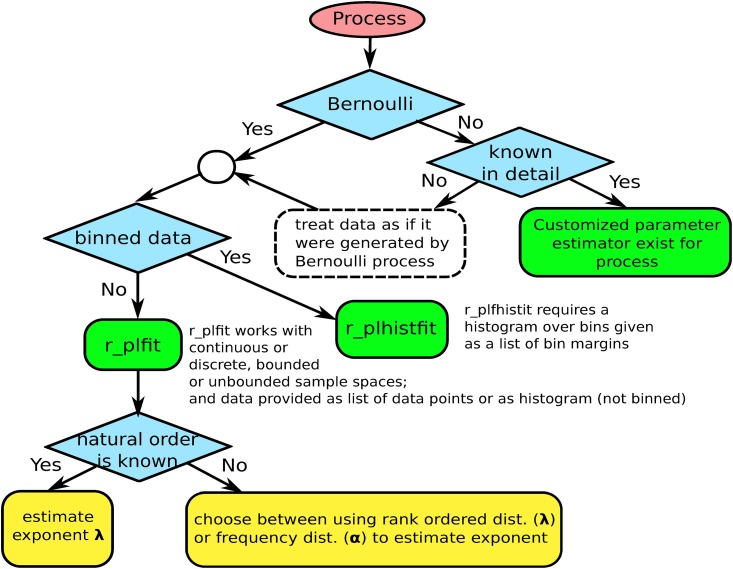
Decision tree of questions that should be clarified before estimating power-law exponents from data. The tree shows under which conditions the fitting algorithms developed in this paper r_plfit and r_plhistfit can be used.

The decisions one has to take before starting to estimate power-law exponents are shown as a decision-tree in (Fig 1). If it is known that the process generating the data is not a Bernoulli process (for example if the process belongs to the family of history dependent processes such as e.g. preferential attachment), then one has the chance to use this information for deriving parameter estimators that are tailored exactly for the particular family of processes. If no such detailed information is available one can only treat the process as if it were a Bernoulli process, i.e. information about correlations between samples is ignored. If we know (or assume) that the data generation process is a Bernoulli process, the next thing to determine is whether the data is available as a collection of data points, or merely as coarse grained information in form of a histogram that collects distinct events into bins (e.g. histograms of logarithmically binned data).

If data is available in form of a data set of samples (not binned), a surprisingly general maximum likelihood (ML) estimator can be used to predict the exponent of an underlying power-law *p*(*x*) ∝ *x*^−λ^. This estimator that we refer to as ML*, will be derived in the main section. Its estimates for the underlying exponent λ, are denoted by λ*. The code for the corresponding algorithm we refer to as r_plfit. If information is available in form of a histogram of binned data, a different estimator becomes necessary. The corresponding algorithm (r_plhistfit) is discussed in S1 File appendix A and in the section below on discrete and continuous sample spaces. Both algorithms are available as matlab code [30]. For how to use these algorithms, see S2 File appendix B.

If we have a dataset of samples (not binned), so that the r_plfit algorithm can be used, it still has to be clarified whether the data has a natural order or not? Numerical observables such as earthquake magnitudes are *naturally* ordered. One earthquake is always stronger or smaller than the other. If observables are non-numeric, such as word types in a text, then a natural order can not be known *a priori*. The natural order can only be inferred approximately by using so-called *rank-ordering*; or alternatively—by using the so-called *frequency distribution* of the data. Details are discussed below in the section on rank-order, frequency distributions, and natural order.

Other issues to clarify are to see if a given sample space is continuous or discrete, and if the sample space is bounded or unbounded. These questions however, turn out to be not critical. One might immediately argue that for unbounded power-law distribution functions normalization becomes an issue for exponents λ ≤ 1. However, this is only true for Bernoulli processes on *unbounded* sample spaces. Since all real-world data sets are collections of finite discrete values one never has to actually deal with normalization problems. Moreover, since most experiments are performed with apparati with finite resolution, most data can be treated as being sampled from a bounded, discrete sample space, or as binned data. For truly continuous processes the probability of two sampled values being identical is zero. Therefore, data sampled from continuous distributions can be recognized by sample values that are unique in a data set. See S1 File appendix A for more details.

Statistically sound ways to fit power-laws were advocated and discussed in [23, 26–29, 31, 32]. They overcome intrinsic limitations of the *least square* (LS) fits to logarithmically scaled data, which were and are widely (and often naively) used for estimating exponents. The ML estimator that was presented in [23] we refer to as the ML_CSN_ (for Clauset-Shalizi-Newman) estimator; its estimates for the exponent we denote by $\hat{\lambda }$. The approach that leads to ML_CSN_ focuses on continuous data *x* that follows a power-law distribution from Eq (1), and that is bounded from below *x* > *x*_min_ > 0 but is not bounded from above (i.e. *x*_max_ > *x* with *x*_max_ = ∞). In [23] emphasis is put on how ML estimators can be used to infer whether an observed distribution function is likely to be a power-law or not. Also the pros and cons of using cumulative distribution functions for ML estimates are discussed, together with ways of treating discrete data as continuous data. For the continuous and unbounded case, simple explicit equations for the ML_CSN_ estimator can be derived [23, 28]. The continuous approach however, even though it seemingly simplifies computations, introduces unnecessary self-imposed limitations with respect to the range of exponents that can be reliably estimated. ML_CSN_ works very well for a range of exponents between −3.5 and −1.5, see Fig (3).

Here we show how to overcome these limitations—and by doing so extend the accessible range of exponents—by presenting the exact methodology for estimating λ for discrete bounded data with the estimator ML*. While this approach appears to be more constrained than the continuous one we can show also theoretically that data from continuous and potentially unbounded sample spaces can be handled within essentially the same general ML framework as well. The key to the ML* estimator is that it is not necessary to derive explicit equations for finding λ*. Implicit equations in λ exist for power-law probability distributions over discrete or continuous sample spaces that are both bounded from below *and* above, see Eq (9), and also [27]. Solutions λ* can be easily obtained numerically. An implementation of the respective algorithms can be found in [30], for a tutorial see S2 File appendix B.

### Rank-order, frequency distributions & natural order

There exist three distinct types of distribution functions that are of interest in the context of estimating power-law exponents:

1. The *probability distribution* *p*(*x*) assigns a probability to every observable state-value *x*. Discrete and bounded sample spaces are characterized by *W* state-types *i* = 1, ⋯, *W*, with each type *i* being associated with a distinct value *x* = *z*_*i*_.
2. The *relative frequencies*, *f*_*i*_ = *k*_*i*_/*N*, where *k*_*i*_ is the number of times that state-type *i* is observed in *N* experiments. *k* = (*k*_1_, ⋯, *k*_*W*_) is the *histogram* of the data. As explained below in detail, the relative frequencies can be ordered in two ways.
  - If *f*_*i*_ is ordered according to their descending magnitude this is called the *rank ordered* distribution.
  - If *f*_*i*_ is ordered according to the descending magnitude of the probability distribution *p*(*z*_*i*_), then they are *naturally ordered* relative frequencies.
3. The *frequency distribution* *ϕ*(*n*) counts how many state-types *i* fulfill the condition *k*_*i*_ = *n*.

**Fig 2:**
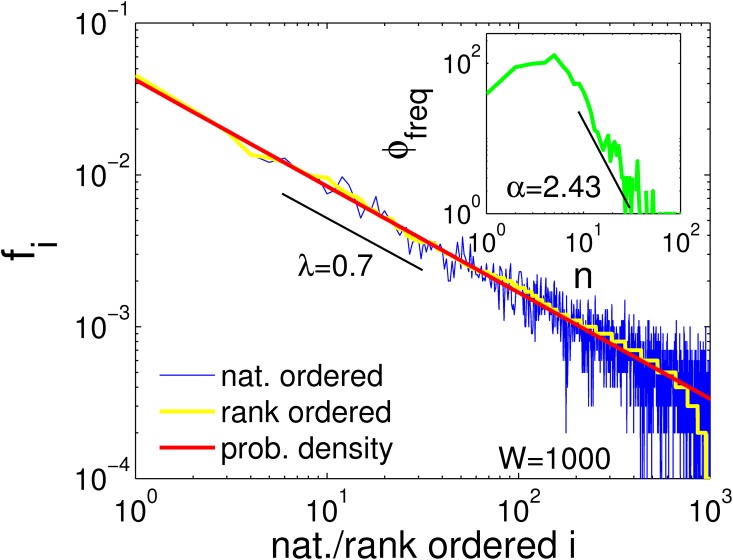
The four types of distribution functions. Data is sampled from a power-law distribution *p*(*x*) ∝ *x*^−λ^ with an exponent λ = 0.7 (red line). The relative frequencies *f*_*i*_ are shown for *N* = 10000 sampled data points according to their natural (prior) ordering that is associated with *p* (blue). The rank-ordered distribution (posterior) is shown in yellow, where states *i* are ordered according to their observed relative frequencies *f*_*i*_. The rank-ordered distribution follows a power-law, except for the exponential decay that starts at rank∼500. A low frequency cut-off should be used to remove this part for estimating exponents. The inset shows the frequency distribution *ϕ*(*n*) that describes how many states *x* appear *n* times (green). The frequency distribution has a maximum and a power-law tail with exponent *α* = 1 + 1/λ ∼ 2.43. To estimate *α*, one should only consider the tail of the frequency distribution function.

In (Fig 2) we show these distribution functions. There *N* = 10000 data points are sampled from *x* ∈ {1, ⋯, 1000}, with probabilities *p*(*x*) ∝ *x*^−0.7^. The probability distribution is shown (red). The relative frequency distribution *f* is plotted in natural order (blue), the rank-ordered distribution is shown with the yellow line, which clearly exhibits an exponential decay towards the the tail. The inset shows the frequency distribution *ϕ*(*n*) of the same data. We next discuss how different sampling processes can be characterized in terms of natural order, rank-order, or frequency distributions.

#### Processes with naturally ordered observables

For some sampling processes the ordering of the observed states is known. For example think of *x* representing the numerical values of earthquake magnitudes. Here any two observations *x* and *x*′ can be ordered with respect to their numerical value, or their *natural order*. Since power-law distributions *p*(*x*) ∝ *x*^−λ^ are monotonic this is equivalent to ranking observations according to the probability distribution *p* they are sampled from: The most likely event has *natural rank* 1, the second most likely rank 2, etc. In other words, we can order state-types *x* in a way that over the sample space Ω = {*z*_*i*_|*i* = 1, ⋯, *W*}, *p* = (*p*(*z*_1_), ⋯, *p*(*z*_*W*_)) is a monotonic and decreasing function.

#### Processes with rank-ordered observables

If *p* is not known *a priori* because the state-types *i* have no numerical values *z*_*i*_ attached, as happens for example with words in a text, we can only count relative frequencies *f*_*i*_ (a normalized histogram) of states of type *i*, *a posteriori*, i.e. after sampling. To be clear, let *k* = (*k*_1_, ⋯, *k*_*W*_) be the histogram of *N* recorded states. *k*_*i*_ is the number of times we observed type *i*, then *f*_*i*_ = *k*_*i*_/*N* is the relative frequency of observing states of type *i*. After all samples are taken, one can now order states with respect to *f*_*i*_, such that the rank 1 is assigned to state *i* with the largest *f*_*i*_, rank 2 to *i*′ with the second largest *f*_*i*′_, etc. *f* = (*f*_1_, ⋯, *f*_*W*_) is called the *rank-ordered* distribution of the data.

The natural order imposed by *p* and the rank-order imposed by *f* are not identical for finite *N*. However, if data points have been sampled independently, then *f* converges toward *p* (for *N* → ∞) and the rank-order induced by *f* will asymptotically approach the natural order induced by *p*. (This is true when data gets sample independently from a fixed distribution, but not in general. If the observable states are not bounded from above as the number of samples increases, then such processes showing emergent power-laws with exponents larger than −1 in rank, cannot be Bernoulli processes. In such cases ML estimators can be derived specifically for the particular sampling process, given the sampling process is known (compare the decision tree in (Fig 1)). If the sampling process is not known, as it often is the case, then one is left with the only option, to treat the data points as if they were sampled independently.) The highest uncertainty on estimating the order induced by *p* using *f* is associated with the least frequent observations. Therefore, when estimating exponents from rank-ordered distributions, one might consider to use a low-frequency cut-off to exclude infrequent data.

#### Frequency distributions

Exponents of power-laws can also be estimated from *frequency* distributions *ϕ*(*n*). These counts how many distinct state-types *i* occur exactly *n* times in the data. It does not depend on the natural (prior) order of states and therefore is sometimes preferred to the (posterior) rank-ordered distribution. However, complications may be encountered for naturally or rank ordered distributions as well as for frequency distributions. If we find a well defined power-like probability distribution *p* ∝ *x*^−λ^ (and asymptotically to *f*), then the associated frequency distribution *ϕ*(*n*) is not an exact power-law, but a non-monotonic distribution (with a maximum). Only the tail of the frequency distribution decays as a power-law, *ϕ*(*n*) ∝ *n*^−*α*^. Conversely, if the frequency distribution is a well defined power-law *ϕ* ∝ *n*^−*α*^, then the probability distribution *p* will not show a well defined power-law *p* ∝ *x*^−λ^ over the entire range of states *x*. As a consequence, one frequently needs to specify the data range for estimating the power-law exponent. The exponents λ and *α* are related through the well known equation [33]

α=1+1/λ. (2)

If the probability distribution has exponent λ, the tail of the associated frequency distribution has exponent *α*. Since the frequency distribution behaves like a power-law only in its tail, estimating *α* makes it necessary to constrain the observed data to large values of *n*. Note that this is equivalent to using a low-frequency cut-off. One option to do that is to derive a maximum entropy functional for *ϕ*(*n*) and fit the resulting (approximate) max-ent solution to the data. We do not follow this route here.

If the natural order of the data is known, one can directly use the natural ordered data in the ML estimates for the exponents. If it is not known, either the rank-ordered distribution can be used to estimate λ, or the frequency distribution to estimate *α*, see (Fig 1).

One might also estimate both, λ in the rank ordered distribution, and *α* in the frequency distribution of the data. Using Eq (2) to compare the two estimates may be used as a rough quality-check. If estimates do not reasonably coincide one should check whether the used data ranges have been appropriately chosen. If large discrepancies remain between *α* and 1 + 1/λ this might indicate that the observed distribution function in question is only an approximate power-law, for which Eq (2) need not hold. For a tutorial on how to use r_plfit to perform estimates see S2 File appendix B.

### Discrete and continuous sample spaces & normalization

Data can originate from continuous sample spaces Ω_*c*_ = [*x*_min_, *x*_max_], or discrete ones Ω_*d*_ = {*z*_1_, *z*_2_, ⋯, *z*_*W*_}. To each state-type *i* = 1, ⋯, *W*, there is assigned a state-value *z*_*i*_. Whether a distribution function $p\left(x\right)=Z_{\lambda }^{-1}x^{-\lambda }$, with *x* ∈ Ω, is normalizable or not, can only be decided once the sample space Ω has been specified. The normalization factors for continuous and discrete Ω are

$$\begin{array}{ccccc} Z_{\lambda }\left(\Omega_{c}\right) & \equiv & \int_{x_{\text{min}}}^{x_{\text{max}}}dx\;x^{-\lambda } & = & \frac{x_{\text{max}}^{1-\lambda }-x_{\text{min}}^{1-\lambda }}{1-\lambda }\\ Z_{\lambda }\left(\Omega_{d}\right) & \equiv & \sum_{x\in \Omega_{d}}x^{-\lambda } & = & \sum_{i=1}^{W}z_{i}^{-\lambda }\;. \end{array}$$ (3)

For bounded sample spaces with 0 < *x*_min_ ≤ *x* ≤ *x*_max_ < ∞, power-laws are always normalizable for arbitrary exponents λ, and a well defined ML estimator of λ* exists (see below). The normalization constants in Eq (3) can be specified in r_plfit (see S2 File appendix B).

Data sampled from a continuous sample space Ω_*c*_ can essentially be treated as if it were sampled from a discrete sample space Ω_*d*_, where *x* ∈ Ω_*d*_ are given by the unique collection of distinct values in the data set. That is, the data set *x* = (*x*_1_, ⋯, *x*_*N*_) contains *N* data points *x*_*n*_ (that have *W* unique values *z*_*i*_, the states of type *i*) which we collect in the discrete sample space Ω = {*z*_1_, ⋯, *z*_*W*_}. For truly continuous data we have *N* = *W*, since the probability of *x*_*m*_ = *x*_*n*_ for *n* ≠ *m* is vanishing. As a consequence the histogram *k*_*i*_, which counts the number of times *z*_*i*_ appears in the data, is essentially given by *k*_*i*_ = 1 for all *i* = 1, ⋯, *W*. This provides us with a practical criterion for when to use the normalization constant for discrete or continuous data. For details see S1 File appendix A.

The equation for the ML estimator ML*, that yields the estimate λ*, only requires the knowledge of the relative frequency distribution *f*_*i*_ = *k*_*i*_/*N* (in natural- or rank-order) of the observed state-types *i*, as we will see in Eq (9) below. Therefore r_plfit can work either with data sets *x* or histograms *k* over the unique values in the data sets. If data comes in coarse grained form, i.e. histograms, where each bin may contain a whole range of observable values *x*, then an estimator is required that is different from ML* [32], see also S1 File appendix A. The corresponding code r_plhistfit can also be downloaded from [30].

## The ML*-estimator for power-laws from discrete sample spaces

Consider a family of random processes *Y* that is characterized by the parameters *θ* = (*θ*_1_, ⋯, *θ*_*R*_). Let *Y* be defined on a discrete sample space Ω = {*z*_1_, *z*_2_, ⋯, *z*_*W*_}, with 0 < *z*_*i*_ < ∞. The process *Y* samples values *x* ∈ Ω with probability,

p(x|θ,Ω). (4)

Let us repeat the process *Y* in *N* independent experiments to obtain a data set *y* = (*y*_1_, ⋯, *y*_*N*_). *k* = (*k*_1_, ⋯, *k*_*W*_) is the histogram of the events recorded in *y*, i.e. *k*_*i*_ is the number of times *z*_*i*_ appears in *y*. Note that $\sum_{i=1}^{W}k_{i}=N$. As a consequence of independent sampling, the probability to sample exactly *k* is,

$$P(k|\theta ,\Omega )=\left({}_{k}^{N}\right)\overset{W}{\underset{i=1}{\prod }}p{\left(z_{i}|\theta ,\Omega \right)}^{k_{i}},$$ (5)

where $\left({}_{k}^{N}\right)=N!/\overset{W}{\underset{i=1}{\prod }}k_{i}!$ is the multinomial factor. Bayes’ formula allows us to get an estimator for the parameters *θ*,

$$P\left(\theta |k,\Omega \right)=P\left(k|\theta ,\Omega \right)\frac{P(\theta |\Omega )}{P(k|\Omega )}\;.$$ (6)

Obviously, *P*(*k*|Ω) = ∫*dθ* *P*(*k*|*θ*, Ω)*P*(*θ*|Ω) does not depend on *θ*. Without further available information we must assume that the parameters *θ* are uniformly distributed between their upper and lower limits. As a consequence, *P*(*θ*|Ω) also does not depend on *θ* within the limits of the parameter range and can be treated as a constant. (Unfortunately, what works for parameters in *θ* such as λ does not work for parameters such as *x*_min_ and *x*_max_. For those variables it turns out that *P*(*θ*|Ω) can not be assumed to be constant between upper and lower bounds of the respective parameter values. Bayesian estimators for *x*_min_ and *x*_max_ require to explicitly consider a non-trivial function *P*(*θ*|Ω). Though in principle feasible, we ignore the possibility of deriving Bayesian estimates for *x*_min_ and *x*_max_ in this paper.) From Eq (6) it follows that the value θ* that maximizes *P*(*θ*|*k*, Ω) also maximizes *P*(*k*|*θ*, Ω). The most likely parameter values θ* = (*θ*_1_, ⋯, *θ*_*R*_)* are now found by maximizing the log-likelihood,

$$\begin{array}{ccc} 0 & = & \frac{\partial }{\partial \theta_{r}}\frac{1}{N}\text{log}P\left(\theta |k,\Omega \right)=\sum_{i=1}^{W}f_{i}\frac{\partial }{\partial \theta_{r}}\text{log}p\left(z_{i}|\theta ,\Omega \right)=-\frac{\partial }{\partial \theta_{r}}H_{\text{cross}}(f||p\left(z|\theta ,\Omega \right))\;, \end{array}$$ (7)

for all parameters *r* = 1, ⋯, *R*. Here $H_{\text{cross}}\left(f||p\left(z|\theta ,\Omega \right)\right)\equiv -\sum_{i=1}^{W}f_{i}\text{log}p\left(z_{i}|\lambda ,\Omega \right)$, is the so-called *cross-entropy*. In other words, ML-estimates maximize the cross-entropy with respect to the parameters *θ*_*r*_.

### The ML*-algorithm for power-laws

To apply Eq (7) for ML-estimates of power-law exponents, one specifies the finite sample space Ω = {*z*_1_, *z*_2_, ⋯, *z*_*W*_}, and the family of probability density functions is,

$$p\left(x|\lambda ,\Omega \right)=\frac{x^{-\lambda }}{Z_{\lambda }\left(\Omega \right)}\;,$$ (8)

with *x* ∈ Ω. Note that the set of parameters *θ* defined above now only contains λ, or *θ* = {λ}. The normalization constant is *Z*_λ_(Ω) = ∑_*x* ∈ Ω_ *x*^−λ^. The derivative with respect to λ of the cross-entropy, $H_{\text{cross}}\left(f||p\left(z|\theta ,\Omega \right)\right)=\lambda \sum_{i=1}^{W}f_{i}\text{log}z_{i}+\text{log}Z_{\lambda }\left(\Omega \right)$, has to be computed, and setting *dH*_cross_/*d*λ = 0 yields

$$\sum_{i=1}^{W}f_{i}\text{log}z_{i}={\left(\sum_{i=1}^{W}z_{i}^{-\lambda }\right)}^{-1}\sum_{i=1}^{W}z_{i}^{-\lambda }\text{log}z_{i}\;.$$ (9)

An equivalent version of this equation is derived in [27] using different means. The solution to this implicit equation, λ = λ*, can not be written in closed form but can be easily solved numerically. See [30] for the corresponding algorithm and S2 File appendix B for a tutorial.

### How to determine λ*

One possibility to find the solution λ = λ* from the implicit equation Eq (9), is to iteratively refine approximate solutions. For this, select *M* + 1 values λ from the interval [λ_min_, λ_max_], where *M* is a finite fixed number, say *M* = 100. Those values may be chosen to be given by the expression

$$\lambda_{r}\left(m\right)={\underline{\lambda }}_{r}+\frac{m}{M}\left({\bar{\lambda }}_{r}-{\underline{\lambda }}_{r}\right)\;,$$ (10)

for *m* = 0, ⋯, *M*. The parameters ${\underline{\lambda }}_{r}$ and ${\bar{\lambda }}_{r}$ are defined in the following way: First define ${\underline{\lambda }}_{1}=\lambda_{\text{min}}$, and ${\bar{\lambda }}_{1}=\lambda_{\text{max}}$, where λ_max_ and λ_min_ are parameters of the algorithm. Then define *δ*λ_1_ = Δλ/*M* with Δλ = λ_max_−λ_min_. If $\lambda_{1}\left(m_{1}^{*}\right)$ is the optimal solution of Eq (9) for some $m_{1}^{*}$, then we can choose ${\underline{\lambda }}_{2}=\lambda_{1}\left(m_{1}^{*}\right)-\delta \lambda_{1}$, and ${\bar{\lambda }}_{2}=\lambda_{1}\left(m_{1}^{*}\right)+\delta \lambda_{1}$ and *δ*λ_2_ = 2*δ*λ_1_/*M*. One then continues by iterating *r* times until *δ*λ_*r*_ < *ε*, where *ε* is the desired accuracy of the estimate of λ*. As a consequence, the value $m_{r}^{*}$, for which $|\lambda^{*}-\lambda_{r}\left(m_{r}^{*}\right)|<\varepsilon$ holds, optimally estimates λ* in the *r*’th iteration with an error smaller than *ε*. Note that *ε* is the error of the ML*-estimator with respect to the exact value of the predictor λ*, and is not the error of λ* with respect to the (typically unknown) value of the exponent λ of the sampling distribution.

Controlling the fit region over which the power-law should be obtained therefore becomes a matter of restricting the sample space to a convenient Ω′ ⊂ Ω. This can be used for dynamically controlling low-frequency cut-offs. These cut-offs are set to exclude states for which,

$$p\left(z_{i}|\lambda ,\Omega \right)N<k_{\text{min}}\;,$$ (11)

where *k*_min_ is the minimal number of times that any state-type *i* is represented in the data set. This means that we re-estimate λ on Ω′ ⊂ Ω with

$$\Omega^{\prime }=\left\{z_{i}\in \Omega |p\left(z_{i}|\lambda ,\Omega \right)N\geq k_{\text{min}}\right\}\;.$$ (12)

We see in Eq (9) that iteratively adapting Ω to subsets Ω′, and then re-evaluating λ, requires to solve,

$$\sum_{i\in I(\Omega^{\prime })}f_{i}^{\prime }\text{log}\left(z_{i}\right)={\left(\sum_{i\in I(\Omega^{\prime })}z_{i}^{-\lambda }\right)}^{-1}\sum_{i\in I(\Omega^{\prime })}z_{i}^{-\lambda }\text{log}z_{i}\;,$$ (13)

where *N*′ = ∑_*i* ∈ *I*(Ω′)_ *k*_*i*_ is the restricted sample-size and $f_{i}^{\prime }=k_{i}/N^{\prime }$ are the relative frequencies re-normalized for Ω′. *I*(Ω′) = {*i*|*z*_*i*_ ∈ Ω′} is the index-set of Ω′.

Iterating this procedure either leads to a fixed point or to a limit cycle between two low-frequency cut-offs with two slightly different estimates for λ*. These two possibilities need to be considered in order to implement an efficient stopping criterion for the iterative search of the desired low-frequency cut-off in the data. The algorithm therefore consists of two nested iterations. The “outer iteration” searches for the low-frequency cut-off, the “inner iteration” solves the implicit equation for the power-law exponent. The matlab code for the algorithm is found in [30], see S2 File appendix B for a tutorial.

## Testing the new estimator with numerical experiments and known data sets

To test the proposed algorithm implementing the estimator ML*, we first perform numerical experiments and then test its performance on a number of well known data sets.

### Testing with numerical experiments

**Fig 3:**
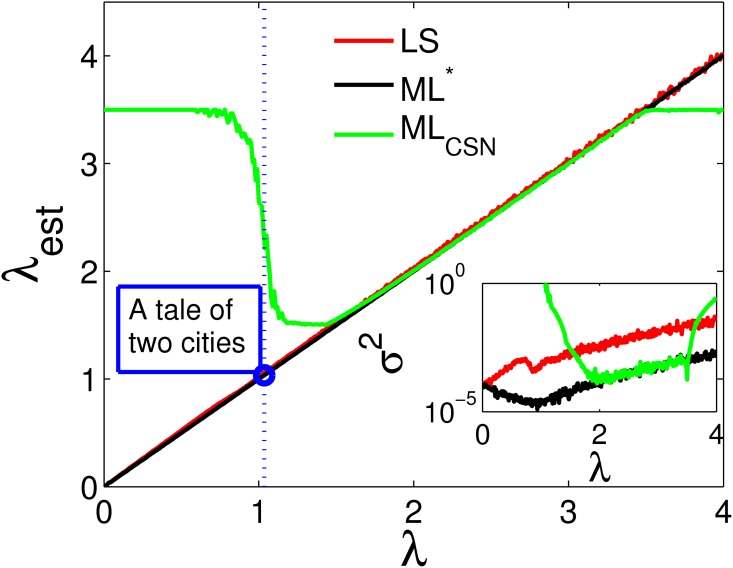
Comparison of the three power-law exponent estimators, LS, ML_CSN_, and ML*. For 400 values of λ in the range between 0 and 4, we sample *N* = 10,000 events from Ω = {1, ⋯, 1,000}, from a power-law probability distribution *p*(*x*|λ, Ω) ∝ *x*^−λ^. The estimated exponents λ_est_ for the estimators LS (red), the ML_CSN_ (green, $\lambda_{\text{est}}=\hat{\lambda }$), and the new ML* (black, λ_est_ = λ*), are plotted against the true value of the exponent λ of the probability distribution samples are drawn from. Clearly, below λ ∼ 1.5 the ML_CSN_ estimator no longer works reliably. ML_CSN_ and ML* work equally well in a range of 1.5 < λ < 3.5. Outside this range ML* performs consistently better than the other methods. The inset shows the mean-square error *σ*^2^ of the estimated exponents. The LS-estimator has a much higher *σ*^2^ over the entire region, than the ML*-estimator. The blue dot represents the ML* estimate for the Zipf exponent of C. Dickens’ “A tale of two cities”. Clearly, this exponent could never reliably be obtained from the rank ordered distribution using ML_CSN_, whereas ML* works fine even for values of λ ∼ 0.

For 400 different values of λ, ranging from 0 to 4, we sample *N* = 10,000 data points *x* ∈ Ω = {1, ⋯, *W*}, with *W* = 1000 states, with probabilities *p*(*x*|λ, Ω) ∝ *x*^−λ^. We fit the data in three ways, using (i) least square fits (LS), (ii) the CSN algorithm ML_CSN_ providing estimates $\hat{\lambda }$, and (iii) the implicit ML* method providing estimates λ*. In Fig 3 we show these estimates for the power exponents, as a function of the true *values* of λ. The LS, ML_CSN_, ML* estimators are shown as the red, green, and black curves respectively. Obviously ML* and ML_CSN_ work equally well for power-law exponents λ with values 1.5 < λ < 3.5. In this range the three approaches coincide. However, note that in the same region the mean square error. (The mean square error is defined as $\sigma^{2}\left(\lambda \right)=N_{\text{rep}}^{-1}\sum_{m=1}^{N_{\text{rep}}}{\left(\lambda_{\text{est}}\left(m\right)-\lambda \right)}^{2}$, where *N*_rep_ is the number of repetitions, i.e. the number of data-sets we sampled from the *p*(*x*|λ, Ω), *x* = 1, …, *W*. λ_est_(*m*) is the value estimated for λ from the *m*th data set. Depending on the estimator λ_est_ corresponds to $\hat{\lambda }$ (ML_CSN_), λ*, (ML*), or the LS estimator. We used *W* = 1000 and *N*_rep_ = 25 for any given λ.) *σ*^2^ for the LS method is much larger than for ML* and ML_CSN_. Outside this range the assumptions and approximations used for ML_CSN_ start to lose their validity and both LS and ML* estimates outperform the ML_CSN_ estimates. The inset also shows that ML* consistently estimates λ much better than the LS estimator (two orders of magnitude better in terms of *σ*^2^) for the entire range of λ. The blue dot in Fig 3 represents the ML* estimate for the Zipf exponent of C. Dickens’ ‘A tale of two cities’. Clearly, this small exponent could never be obtained by ML_CSN_, see also Table 1.

**Table 1:** Comparison of the estimators ML* and ML_CSN_ on empirical data sets that were used in [23]. These include the frequency of surnames, intensity of wars, populations of cities, earthquake intensity, numbers of religious followers, citations of scientific papers, counts of words, wealth of the Forbes 500 firms, numbers of papers authored, solar flare intensity, terrorist attack severity, numbers of links to websites, and forest fire sizes. We added the word frequencies in the novel “A tale of two cities” (C. Dickens). The second column states if *α* or λ were estimated. The exponents reported in [23] are found in column CSN_1_, those reproduced by us applying their algorithm to data [23, 34–37] is shown in column CSN_2_. The latter correspond well with the new ML* algorithm. For values λ < 1.5, CSN can not be used. We list the corresponding values for Kolmogorov-Smirnov test for the two estimators, KS_CSN_ and KS*.

	exp.	CSN_1_	CSN_2_	ML*	KS_CSN_	KS*
blackouts	λ	2.3	2.27	2.25	0.061	0.031
surnames	*α*	2.5	2.49	2.66	0.041	0.019
int. wars	λ	1.7	1.73	1.83	0.078	0.076
city pop.	λ	2.37	2.36	2.31	0.019	0.016
quake int.	λ	1.64	1.64	1.88	0.092	0.085
relig. fol.	λ	1.8	1.79	1.61	0.091	0.095
citations	λ	3.16	3.16	3.10	0.010	0.018
words	*α*	1.95	1.95	1.99	0.009	0.015
wealth	λ	2.3	2.34	2.30	0.063	0.066
papers	λ	4.3	4.32	3.89	0.079	0.082
sol. flares	λ	1.79	1.79	1.81	0.009	0.021
terr. attacks	λ	2.4	2.37	2.36	0.018	0.017
websites	λ	2.336	2.12	1.72	0.025	0.056
forest fires	λ	2.2	2.16	2.46	0.036	0.034
Dickens novel	λ	-	-	1.04	-	0.017

### Testing with empirical data sets

We finally compare the new estimator ML* on several empirical data sets that were used for demonstration in [23]. In Table 1 we collect the results. The second column states if λ or *α* were estimated. Column CSN_1_ presents the value of the estimator ML_CSN_ as presented in [23]. Column CSN_2_ contains the values of the same estimator using the data from [23] and using the algorithm provided by [31]. (The reason for the differences might be that some of the data has been updated since the publication.) The results for the ML* estimator agrees well with those of ML_CSN_ in the range where the latter works well. To demonstrate how ML* works perfectly outside of the comfort zone of ML_CSN_ (for λ < 1.5), we add the result of the rank distribution of word counts in the novel “A tale of two cities” (Charles Dickens, 1859), which shows an exponent of λ ∼ 1.035. This exponent can be fitted directly from the data using the proposed ML* algorithm, while ML_CSN_ can not access this range, at least not without the detour of first producing a histogram from the data and then fitting the tail of the frequency distribution. The values for the corresponding Kolmogorov-Smirnov tests (see e.g. [23]) for the two estimates, KS_CSN_ and KS*, are similar for most cases.

## Conclusions

We discuss the generic problem of estimating power-law exponents from data sets. We list a series of questions that must be clarified before estimates can be performed. We present these questions in form of a decision tree that shows how the answers to those questions lead to different strategies for estimating power-law exponents.

To follow this decision tree can be seen as a recipe for fitting power exponents from empirical data. The corresponding algorithms were presented and can be downloaded as matlab code. The two algorithms we provide are based on a very general ML estimator that maximizes an appropriately defined cross entropy. The method can be seen as a straight forward generalization of the idea developed in [23]. The two estimators (one for binned histograms and ML* for raw data sets) allow us to estimate power-law exponents in a much wider range than was previously possible. In particular, exponents lower than λ < 1.5 can now be reliably obtained.

## Supporting information

S1 File APPENDIX A: Sampling from continuous sample spaces. Provides a short discussion on ML estimates performed on data sampled from continuous sample spaces.
(PDF)

S2 File APPENDIX B: Using r_plfit. Gives a short introduction on how to use the matlab implementations of the algorithms r_plfit and r_plhistfit.
(PDF)

S3 File APPENDIX C: The false rejection rate of power-laws. Discusses the *p*-value of the method and how the *p*-values obtained from the KS goodness of fit test can be interpreted. It also includes a figure that exemplifies how r_plfit_calib_eval.m can be used to visualize calibration curves sampled with r_plfit_calibrate.m, in order to control the false rejection rate (*p*-value) of r_plfit.m.
(PDF)

S4 File APPENDIX D: Code. Contains the code for all the m-files in print form. Alternatively to the download we provide, [30], the code can be accessed using copy-and-paste.
(PDF)
