# Correction: Fitting power-laws in empirical data with estimators that work for all exponents

**DOI:** 10.1371/journal.pone.0196807

**Published:** 2018-04-27

**Authors:** Rudolf Hanel, Bernat Corominas-Murtra, Bo Liu, Stefan Thurner

In the Funding section, the grant number from the funder Austrian Science Foundation “Fonds zur Förderung der wissenschaftlichen Forschung” is listed incorrectly. The correct grant number is: P29032-N32.
