# Supplementary material for: Fitting power-laws in empirical data with estimators that work for all exponents
Source: PLoS One. 2017 Feb 28;12(2):e0170920. doi: 10.1371/journal.pone.0170920 (PMC5330461; doi:10.1371/journal.pone.0170920)
Supplement: S1 File — Provides a short discussion on ML estimates performed on data sampled from continuous sample spaces. (PDF) [file pone.0170920.s001.pdf]

S1: Fitting power-laws in empirical data with estimators that work for all exponents

Rudolf Hanel[1], Bernat Corominas-Murtra[1], Bo Liu[1], Stefan Thurner[1,2,3,4],

1 Section for Science of Complex Systems, Medical University of Vienna, Spitalgasse 23, 1090 Vienna, Austria
2 Santa Fe Institute, 1399 Hyde Park Road, Santa Fe, NM 87501, USA
3 IIASA, Schlossplatz 1, 2361 Laxenburg, Austria
4 Complexity Science Hub Vienna, Josefstädterstrasse 39, A-1090 Vienna, Austria

¤Current Address: Section for Science of Complex Systems, CeMSIIS, Medical University of Vienna, Spitalgasse 23, Bauteil 86, A-1090, Vienna, Austria
* stefan.thurner@meduniwien.ac.at

APPENDIX A: Sampling from continuous sample spaces

If events x are drawn from a continuous sample space $\Omega = [x_{\rm min},\, x_{\rm max}]$, for instance the magnitude of earthquakes, then the 'natural order' of possible events is simply given by the magnitude x of the observation. Events x are drawn from a continuous power-law distribution $p(z|\lambda, \Omega) = x^{-\lambda}/Z$, with $Z = Z_\lambda([x_{\rm min},\, x_{\rm max}])$ (compare Eq. (main 3) first line).

To work with well defined probabilities we have to bin the data first. Probabilities to observe events within a particular bin depend on the margins of the W bins $b = (b_0, b_1, \cdots, b_W)$, with $b_0 = x_{\rm min}$ and $b_W = x_{\rm max}$. The histogram $k = (k_1, \cdots, k_W)$ counts the number k_i of events x falling into the bin $b_i > x \geq b_{i-1}$, and the probability of observing x in the i'th bin is given by

$$p(i|\lambda, x) = \frac{b_i^{1-\lambda} - b_{i-1}^{1-\lambda}}{x_{\rm max}^{1-\lambda} - x_{\rm min}^{1-\lambda}} \quad . \tag{1}$$

Binning events sampled from a continuous distribution may have practical reasons. For instance data may be collected from measurements with different physical resolution levels, so that binning should be performed at the lowest resolution of data points included in the collection of samples. We will not discuss the ML estimator for binned data in detail but only remark that for given bin margins b it is sufficient to insert $p(i|\lambda, x)$ of Eq (1) into Eq. (main 7) with $\theta = \{\lambda\}$, to derive the appropriate ML condition for binned data. An algorithm for binned data `r_plhistfit`, where we assume the bin margins b_i to be given, is found in [2].

We point out that if margins for binning have not been specified prior to the experiments, then specifying the optimal margins for binning the data becomes a parameter estimation problem in itself, i.e. the optimal margins b_i have to be estimated from the data as well. One major source of uncertainty in the estimates of λ from binned data is related to the uncertainty in choosing the upper and lower bounds $x_{\rm min}$ and $x_{\rm max}$ of the data, i.e. specifying the bounds of the underlying continuous sample space.

Binning becomes irrelevant for clean continuous data for the following reason. Suppose we fix the sample space $[x_{\rm min},\; x_{\rm max}]$ and cut this domain into M bins of width

$\Delta = (x_{\max} - x_{\min})/M$. Since the data $x = \{x_1, \cdots, x_N\}$ is drawn from a continuous sample space, the chance for two observations x_m and x_n to be exactly equal becomes zero for $m \neq n$, if M has been chosen sufficiently large. Then each bin almost certainly contains either one sample x_n or none. The probability of observing x then is asymptotically (as Δ approaches zero) given by

$$P(x|\lambda) = \Delta^N \prod_{n=1}^{N} \left(\frac{x_n^{-\lambda}}{Z_\lambda(x_{\min}, x_{\max})} \right) \quad . \tag{2}$$

The parameter estimation problem of finding the optimal λ is equivalent to maximizing $P(x|\lambda)$ (or equivalently $\log P(x|\lambda)$) with respect to λ. In this maximization problem Δ becomes irrelevant and only the choice of $x_{\min}$ and $x_{\max}$ and the data x remains relevant for the estimate. As a consequence, one obtains an equation

$$\sum_{i=1}^{W} f_i \log z_i = \frac{d}{d\lambda} \log Z_\lambda \, , \tag{3}$$

for the ML estimate of the exponent λ over continuous sample spaces. Equation (main 9) and Eq. (3) differ only in Z_λ. In Eq. (main 9) the normalization constant of discrete samples spaces gets used while in Eq. (3) Z_λ is the normalization constant for a continuous sample space. Switching between continuous and discrete sample spaces therefore is simply a matter of choosing the one or the other normalization constant in the algorithm.

Whether data should be assumed to be sampled from continuous or discrete sample spaces is not always totally clear. Many measurements have an intrinsic resolution and implicitly bin the data. For instance if real numbers sampled in an experiment are given only with a three digit precision, such as $x_n = 0.123$ and we know that $0.001 = x_{\min}$ and $x_{\max} = 5$ then we better treat the data as discrete data on $\Omega_d = \{0.001, 0.002, \cdots, 4.998, 4.999, 5\}$ if we have sufficiently many samples for the histogram over Ω_d not to be flat. A primitive test to see whether one should regard data as sampled from a continuous sample space or not is to make a histogram over the unique values of the recorded data. If each distinct value appears only once in the data (i.e. if the histogram over the unique data-points is flat) then one should treat the sample-space as continuous.

While for the discrete case we need not estimate $x_{\min}$ and $x_{\max}$ this remains necessary for the continuous case. The method of cutting the $[x_{\min}, x_{\max}]$ into segments of length Δ and then taking Δ to zero explains why typically tha *primitive* estimates, $x_{\min} = \min\{x_n | n = 1, \cdots, N\}$ and $x_{\max} = \max\{x_n | n = 1, \cdots, N\}$, provides fairly good results. Alternatively, strategies such as suggested in [1] could be used to optimize the choices for $x_{\min}$ and $x_{\max}$. However, this procedure can not be directly derived from Bayesian arguments. Neither will we discuss this approach in this paper nor implement such an option in `r_plfit`.

However, Bayesian estimates of $x_{\min}$ and $x_{\max}$ exist. Although we will not discuss those estimators in detail here we will eventually implement them in `r_plfit` to replace the primitive estimates. The idea of constructing such estimators is the following. For instance, one asks how likely can the maximal value $\max(x)$ of the sampled data $x = (x_1, \cdots, x_N)$ be found to be larger than some value y. By deriving $P(\max(x) > y | \lambda, [x_{\min}, x_{\max}])$ and $P(\min(x) < y | \lambda, [x_{\min}, x_{\max}])$, as a consequence, it becomes possible to derive Bayesian estimators for $x_{\min}$ and $x_{\max}$.

References

1. Clauset A, Shalizi CR, and Newman MEJ (2009) Power-Law Distributions in Empirical Data, SIAM Rev **51** 661–703.

2. http://www.complex-systems.meduniwien.ac.at/SI2016/r_plfit.m
   http://www.complex-systems.meduniwien.ac.at/SI2016/r_plhistfit.m
   http://www.complex-systems.meduniwien.ac.at/SI2016/r_randi.m
   http://www.complex-systems.meduniwien.ac.at/SI2016/r_plfit_calibrate.m
   http://www.complex-systems.meduniwien.ac.at/SI2016/r_plfit_calib_eval.m
   Alternatively, see also S4 File Appendix D for the code.
