# Supplementary material for: Fitting power-laws in empirical data with estimators that work for all exponents
Source: PLoS One. 2017 Feb 28;12(2):e0170920. doi: 10.1371/journal.pone.0170920 (PMC5330461; doi:10.1371/journal.pone.0170920)
Supplement: S2 File — Gives a short introduction on how to use the matlab implementations of the algorithms r_plfit and r_plhistfit. (PDF) [file pone.0170920.s002.pdf]

S2: Fitting power-laws in empirical data with estimators that work for all exponents

Rudolf Hanel[1], Bernat Corominas-Murtra[1], Bo Liu[1], Stefan Thurner[1,2,3,4],

1 Section for Science of Complex Systems, Medical University of Vienna, Spitalgasse 23, 1090 Vienna, Austria
2 Santa Fe Institute, 1399 Hyde Park Road, Santa Fe, NM 87501, USA
3 IIASA, Schlossplatz 1, 2361 Laxenburg, Austria
4 Complexity Science Hub Vienna, Josefstädterstrasse 39, A-1090 Vienna, Austria

¤Current Address: Section for Science of Complex Systems, CeMSIIS, Medical University of Vienna, Spitalgasse 23, Bauteil 86, A-1090, Vienna, Austria
* stefan.thurner@meduniwien.ac.at

APPENDIX B: Using r_plfit

The matlab function

```
function out = r_plfit(data,varargin)
```

implements the algorithm discussed in the main paper. The function returns a struct `out` that contains information about the data, the data range, but most and for all `out.exponent` returns the estimated exponent of the power-law. Whether the exponent `out.exponent` is the exponent λ of the sample distribution or the exponent α of the frequency distribution of the data depends on how `function out = r_plfit(data,varargin)` gets used as explained below. In the code the sample space Ω is equivalent to a vector $z = [z_1, \cdots, z_W]$ containing W distinct event magnitudes z_i, $i = 1, \cdots, W$.

The variable `data` can be used to import data while a variable number of arguments can be set by `varargin` to tell the algorithm which type of data it should handle and to control the range of the data. By default the only argument that has to be set is `data`. `r_plfit` filters data from data points `data<=0, NaN, Inf`. The data passed on to `data` can be

- a vector of observations `data` $\equiv x = [x_1, \cdots, x_N]$ (default)
- a histogram `data` $\equiv k = [k_1, \cdots, k_W]$ of recorded event types $i = 1, \cdots, W$

`out = r_plfit(data,varargin)` can be used in three basic modes

- `out = r_plfit(x)` returns the estimated exponent λ of the **probability distribution** given the observation x (default)
- `out = r_plfit(k,'hist')` returns the estimated exponent λ of the **probability distribution** given the histogram of observations k
- `out = r_plfit(k)` returns the estimated exponent α of the **frequency distribution** given the histogram of observations k

The third mode `out = r_plfit(k)` is in fact identical to the first mode `out = r_plfit(x)`, only that passing a histogram as sample data to the algorithm is identical to asking how many of the W states i have been observed n times. But this is exactly the frequency distribution of the process, which possesses a tail with exponent $\alpha = 1 + 1/\lambda$. Depending on the mode `r_plfit` returns the exponent λ or α in `out.exponent`

Fitting with observations x: If we run `out = r_plfit(x)` without further options `r_plfit` assumes by default that the data x consists of natural numbers, and that the process samples have been sampled from the sample space $\Omega = \{\min(x), \min(x)+1, \cdots, \max(x)-1, \max)\}$, i.e. $\min(x) \leq z_i = i \leq \max(x)$. If this is not the case one can either specify the data range using all W *unique* values $z = [z_1, \cdots, z_W]$ occurring in the data x by using the option `out = r_plfit(x,'urange')`. In order to define a fit range maximal and minimal data values taken into account can be set by `out = r_plfit(x,'urange','rangemin',minval, ... ... 'rangemax',maxval)` such that `r_plfit` only takes into account data in the range `minval` $\leq z \leq$ `maxval`. To control the data range individually use `out = r_plfit(x,'range',z)`. If the data has been sampled from a continuous sample space, and the histogram over the unique data is flat, i.e. each value in the data only appears once (more or less), then one can tell `r_plfit` that the data is sampled from a continuous sample space by setting the option `'cdat'`, i.e. by running `out = r_plfit(x,'cdat', ...)`. This option tells the algorithm to use the normalization constant for continuous sample spaces and estimates $x_{\min} = \min(x)$ and $x_{\max} = \max(x)$. Moreover, `'cdat'` implicitly sets the `'urange'` and the `'nolf'` option. `'nolf'` (see below) switches off the search of the algorithm for an optimal low frequency cut-off.

Fitting with histograms k: Using histograms k as input works in exactly the same way as for fitting x if we want to estimate the exponent α of the frequency distribution and use `r_plfit` in the `out = r_plfit(k)` mode. If we use `r_plfit` in the `out = r_plfit(k,'hist')` mode, the algorithm assumes by default that the sample space z is given by $z = [1, 2, \cdots, W]$. The option `'urange'` has no effect in this mode and gets ignored if set. Otherwise one can again use the `'range'` property to set the event magnitudes z (the sample space) using `out = r_plfit(k,'hist','range',z)`. The `'minrange'` and `'maxrange'` options work in exactly the same way as before.

Dynamic low frequency cut-off: By default `r_plfit(data)` runs an iterative search for an optimal low frequency cut-off that is set at a range value z_i such that the expected number of samples for z_i equals the variable $N_{\min}$ (default value 1, reset using option `'Nmin'`). This means the algorithm performs a low frequency cut-off for observations x. If however `maxval` is smaller than the predicted cut-off then the low frequency cut-off has no effect. One should note that in the mode `out = r_plfit(k)` the low frequency cut-off mechanism effectively acts as a high frequency cut-off with respect to the data x. One can switch this mechanism off by setting the option `'nolf'` (no low frequency cut-off).

The `'plot'` option, `out = r_plfit(data,... ...,'plot')`, can be used for visualization. `r_plfit` plots the fit over the data in double logarithmic coordinates (loglog plot). Using the option `'figure'` behaves like `'plot'` but explicitly opens a new figure. `'exp_min'` can be used to specify the minimal search value for the exponents (default is 0) and `'exp_max'` to set the maximal search value (default is 5). `'eps'` can be used to set the precision of the implicit algorithm (default $1e-5$). Several other options exist to control the performance of the algorithm, which all can be listed by using `r_plfit('help')` in the command line, which prints a brief manual on the usage of `r_plfit` and available options.

The struct `out` produced by `r_plfit` contains information on the parameters used

by the ML* estimator. The variable `out.exponent` returns the estimated exponent. `r_plfit` performs a Kolmogorov-Smirnov (KS) goodness of fit test (GOF) at a default confidence level of 0.05. This level can be altered set to `level` using the option `r_plfit(...,'KSlevel',level,...)`. The KS test has been implemented using the built in matlab function `kstest2`. The flag `out.KSH` is 0 if the power-law hypothesis should be accepted according to the KS GOF-test, and `out.KSH` is 1 if the power-law hypothesis should be rejected. `out.KSP` returns the p-value of the KS GOV-test. Note that the power-law hypothesis needs to be rejected according to the KS test if the associated p-value, `out.KSP`, is *smaller* than the confidence level. `out.KSS` returns the KS value estimated by `kstest2`.

However, we need to point out that the KS GOF-test is not telling us much about whether or not the estimated data has been generated by a power-law. In fact, to control the false rejection rate, which is what a p-value is good for, one needs to know the p-values of the entire ML^* estimator (see S3 File APPENDIX C).

Using r_plhistfit

If one works with binned data, e.g. histogram data counting the number of events falling into exponentially scaled bins (log-binning), then `r_plhistfit` needs to be used instead of `r_plfit`. The function `function out = r_plhistfit(data,varargin)` like `r_plfit`, by default, uses only data as input and other variables can be set optionally. `data` is always a histogram k that is a vector $k = [k_1, \cdots, k_W]$. Bins can be specified by giving bin margins $b = [b_0, b_1, \cdots, b_W]$ such thatevents counted in k_i had a magnitude x such that $b_{i-1} \leq x < b_i$. Usage, `r_plhistfit(k,'margins',b)`. By default `r_plhistfit` assumes that $b_i = i + 1/2$. Other options work similar to the ones available for `r_plfit` and can be reviewed by typing `r_plhistfit('help')` in the matlab command line.
