# Supplementary material for: Fitting power-laws in empirical data with estimators that work for all exponents
Source: PLoS One. 2017 Feb 28;12(2):e0170920. doi: 10.1371/journal.pone.0170920 (PMC5330461; doi:10.1371/journal.pone.0170920)
Supplement: S3 File — Discusses the p-value of the method and how the p-values obtained from the KS goodness of fit test can be interpreted. It also includes a figure that exemplifies how r_plfit_calib_eval.m can be used to visualize calibration curves sampled with r_plfit_calibrate.m, in order to control the false rejection rate (p-value) of r_plfit.m. (PDF) [file pone.0170920.s003.pdf]

# S3: Fitting power-laws in empirical data with estimators that work for all exponents

Rudolf Hanel[1], Bernat Corominas-Murtra[1], Bo Liu[1], Stefan Thurner[1,2,3,4],

**1** Section for Science of Complex Systems, Medical University of Vienna, Spitalgasse 23, 1090 Vienna, Austria
**2** Santa Fe Institute, 1399 Hyde Park Road, Santa Fe, NM 87501, USA
**3** IIASA, Schlossplatz 1, 2361 Laxenburg, Austria
**4** Complexity Science Hub Vienna, Josefstädterstrasse 39, A-1090 Vienna, Austria

¤Current Address: Section for Science of Complex Systems, CeMSIIS, Medical University of Vienna, Spitalgasse 23, Bauteil 86, A-1090, Vienna, Austria
* stefan.thurner@meduniwien.ac.at

## APPENDIX C: The false rejection rate of power-laws

The KS goodness of fit (GOF) test is not actually testing whether the estimated data has been generated by a power-law or not. It estimates the false rejection rate of power-laws with respect to the estimated exponent. Since the exponent of a power-law is measured with a finite accuracy the KS GOF-test tells you whether the estimated exponent is acceptable rather than measuring whether the hypothesis that what we observe is a power-law or not. To control the false rejection rate of the power-law hypothesis, which is what a p-value is good for, one needs to know the p-values of the entire $ML^*$ estimator.

Let $KS$ be the same variable,

$$\mathrm{KS} = \max_{i\in\mathrm{range}} \left\{ |F_{\mathrm{data}}(i) - F_\alpha(i)| \right\} , \tag{1}$$

that is used in the statistics of the KS GOF-test, where $F_{\mathrm{data}}(i)$ is the cumulative distribution-function generated from the data (the cumulative of the normalized histogram), and $F_\alpha(i)$ is the cumulative distribution function with regard to the estimated exponent $\alpha$. By sampling a large number of data-sets from exact power-laws and looking at the distribution of corresponding KS values, measuring the deviation between the power-law with estimated exponent and the data, one obtains the p-values of the $\mathrm{ML}^*$ estimator.

We provide an algorithm `r_plfit_calibrate`, and `r_plfit_calib_eval`, which can be used to determine the critical value $\mathrm{KS}_{\mathrm{crit}}$ such that rejecting an $ML^*$ estimate with $\mathrm{KS} \geq \mathrm{KS}_{\mathrm{crit}}$ and accepting estimates $\mathrm{KS} < \mathrm{KS}_{\mathrm{crit}}$ allows us to control the actual false rejection rate of the $ML^*$ estimator. I.e. if we calibrate $\mathrm{KS}_{\mathrm{crit}}$ for a expected exponent $\alpha$, a given sample size, and a given confidence level, e.g. the confidence level 0.05, then the resulting value $\mathrm{KS}_{\mathrm{crit}}$ ensures that if we sample from an exact power-law with exponent $\alpha$, we will reject only 5% of all the sampled data. In contrast to what one may expect from the KS-GOF test $\mathrm{KS}_{\mathrm{crit}}$ becomes rather large and many data sets that would be rejected by the KS-GOF test need in fact to be accepted!

The calibration algorithm,
`out1 = r_plfit_calibrate(alpha,W, Nsamples, Nrep)`, requires the variables `alpha`, the expected exponent, `W`, the number of states found in the sample, `Nsamples`,

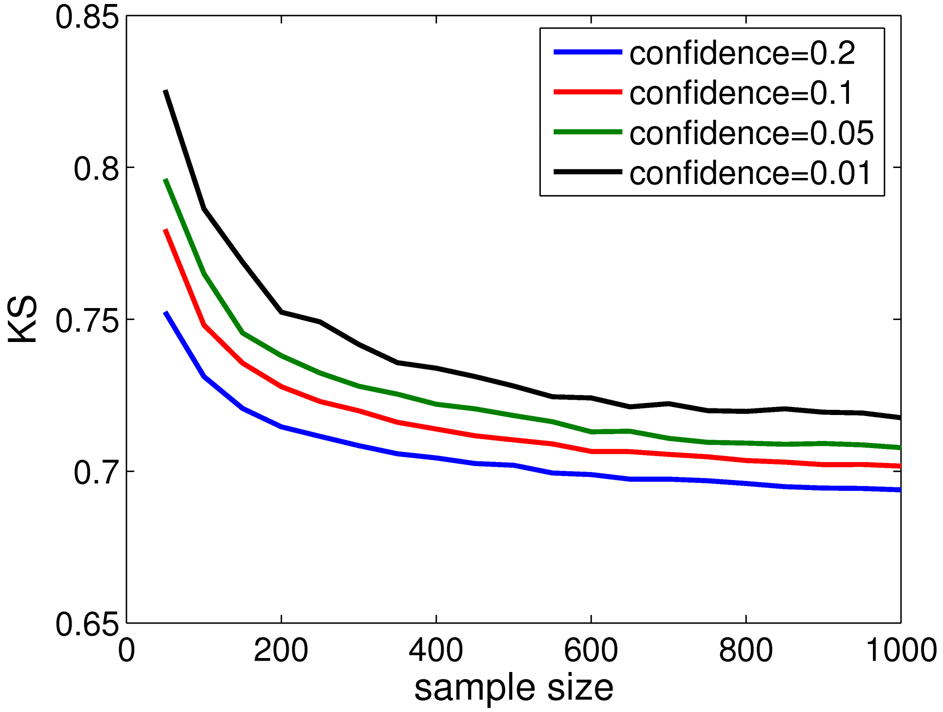


**Fig 1.** Calibration curves for an expected exponent $\alpha = 1$ and $W = 100$ states. Depending on the sample-size the critical KS values are shown for confidence levels 0.01, 0.05, 0.1 and 0.2. Curves have been computed using $\mathrm{out1} = \mathrm{r\_plfit\_calibrate}(\alpha, W, \mathrm{Nsamples}, 1000)$, with $\mathrm{Nsamples} = 50 : 50 : 1000$, and a function to evaluate the calibration data, $\mathrm{out2} = \mathrm{r\_plfit\_calib\_eval}(out1, p, N, 1)$. The critical threshold values $\mathrm{KS_{crit}}$, of the KS parameter for a sample size $N = 500$ are given by 0.7245 (confidence $p = 0.01$), 0.7163 ($p = 0.05$), and 0.7090 ($p = 0.1$), and 0.6994 ($p = 0.2$).

the a vector of sample sizes, e.g. `Nsamples=(500:500:25000)`, and `Nrep`, the number of times we sample a sample of size `Nsamples` from an exact power-law distribution with exponent `alpha`. Typically `Nrep` of order 1000 suffices to get good estimates for the critical p-values of the $ML^*$ estimator. After running `r_plfit_calibrate`, which may take some time, one can use
`out2 = r_plfit_calib_eval(out1,confidence,samplesize,plotflg)` to obtain $\mathrm{KS_{crit}}$ which is returned as `out2.KScrit` by `r_plfit_calib_eval` for the confidence level `confidence` and the sample-size `samplesize` within the range specified in `Nsamples`. The flag `plotflg` can be used for plotting calibration curves ($plotflg = 1$ or $plotflg = 2$) or suppressing the plot ($plotflg = 0$).

Figure (1) shows examples of calibration curves for $\alpha = 1$ and sample sizes in the range of $N = 50$ to $N = 1000$. It becomes obvious that the negative rejection rate is critically controlled by large KS values ($> 0.65$). The maximal possible value is KS$= 1$. This paints a very different picture than we might expect from the KS-GOF test, which rejects hypothesis at much smaller values of the KS statistics. This means that calibrating the false rejection rate of the power-law hypothesis is one thing. Whether the estimate of $\alpha$ is good enough for the KS-GOF test to accept that the data has been sampled from a power-law with exactly the estimated exponent is a totally different question. We therefore can use the calibrated KS values to accept whether or not we

believe data to be sampled from a power-law and we may rely on the KS goodness of fit test whether or not to believe in the exponent we have estimated.
