# Supplementary material for: Fitting power-laws in empirical data with estimators that work for all exponents
Source: PLoS One. 2017 Feb 28;12(2):e0170920. doi: 10.1371/journal.pone.0170920 (PMC5330461; doi:10.1371/journal.pone.0170920)
Supplement: S4 File — Contains the code for all the m-files in print form. Alternatively to the download we provide, [30], the code can be accessed using copy-and-paste. (PDF) [file pone.0170920.s004.pdf]

S4: Fitting power-laws in empirical data with estimators that work for all exponents

Rudolf Hanel[1], Bernat Corominas-Murtra[1], Bo Liu[1], Stefan Thurner[1,2,3,4],

1 Section for Science of Complex Systems, Medical University of Vienna, Spitalgasse 23, 1090 Vienna, Austria
2 Santa Fe Institute, 1399 Hyde Park Road, Santa Fe, NM 87501, USA
3 IIASA, Schlossplatz 1, 2361 Laxenburg, Austria
4 Complexity Science Hub Vienna, Josefstädterstrasse 39, A-1090 Vienna, Austria

¤Current Address: Section for Science of Complex Systems, CeMSIIS, Medical University of Vienna, Spitalgasse 23, Bauteil 86, A-1090, Vienna, Austria
* stefan.thurner@meduniwien.ac.at

APPENDIX D: Code

For printing the code all unnecessary comments have been removed.

APPENDIX D1: r_plfit

```
%%%%%%%%%%%%%%%%%%%%%%%%%%%%%%%%%%%%%%%%%%%%%%%%%%%%%%%%%%%%%%%%%%%%%%%%
% Author: R Hanel: 1.6.2016; last modification on 10.08.2016
%%%%%%%%%%%%%%%%%%%%%%%%%%%%%%%%%%%%%%%%%%%%%%%%%%%%%%%%%%%%%%%%%%%%%%%%
function out = r_plfit(z,varargin)
%%%%%%%%%%%%%%%%%%%%%%%%%%%%%%%%%%%%%%%%%%%%%%%%%%%%%%%%%%%%%%%%%%%%%%%%
% Main begin
%%%%%%%%%%%%%%%%%%%%%%%%%%%%%%%%%%%%%%%%%%%%%%%%%%%%%%%%%%%%%%%%%%%%%%%%
if length(z)==length('help'), if strcmp(z,'help')==1,
        msg=['Using function out = r_plfit(data,varargin)\n'];
  msg=[msg 'r_plfit can be used to fit the exponent out.exponent of power
      laws\n'];
  msg=[msg 'in three basic different modes\n'];
  msg=[msg '(1) out = r_plfit(x) with data being samples x=[x1,...,xN]
      from N experiments\n'];
  msg=[msg '\t in this mode out.exponent returns the estimated exponend
      lambda of \n'];
  msg=[msg '\t the distribution p(z_i|lambda) one has sampled from\n'];
  msg=[msg '(2) out = r_plfit(k,''hist'') with data being histograms
      k=[x1,...,xW]\n'];
  msg=[msg '\t of W distinct events i=1..W with magnitudes
      z=[z1,...,zW]\n'];
  msg=[msg '\t here out.exponent also returns lambda\n'];
  msg=[msg '(3) out = r_plfit(k) with data being histograms
      k=[x1,...,xW]\n'];
  msg=[msg '\t works equivalent to (1) but with k as data one computes
      the\n'];
  msg=[msg '\t frequency distribution of x with exponent
      alpha~1+1/lambda\n'];
  msg=[msg '\n'];
  msg=[msg 'Fitting with data x:\n'];
  msg=[msg '\t By default r_plfit(x) assumes that x consists of natural
      numbers and\n'];
  msg=[msg '\t the sample space (magnitudes) z={i|min(x)<=i<=max(x)}\n'];
  msg=[msg '\t if magnitudes z_i are not of this form one either uses\n'];
```

```
msg=[msg '\t if magnitudes z_i are not of this form one either uses:\n'];
msg=[msg '\t out = r_plfit(x,''urange'') ... which sets z=[z_1,...,z_W]=unique(x)\n'];
msg=[msg '\t where W is then the number of unique values of data points in x\n'];
msg=[msg '\t out = r_plfit(x,''urange'',''rangemin'',zmin,''rangemax'',zmax) can be used\n'];
msg=[msg '\t to specify a fit range zmax <= z_i <= zmin (default zmin=min(x), zmax=max(x))\n'];
msg=[msg '\t To control the data range individually use out = r_plfit(x,''range'',z)\n'];
msg=[msg '\n'];
msg=[msg 'Fitting with histograms k\n'];
msg=[msg '\t Using k (mode 3) for fitting the frequenvcy distribution with exponent alpha\n'];
msg=[msg '\t works in exactly the same way as the sample distribution estimating the\n'];
msg=[msg '\t exponent lambda using x.\n'];
msg=[msg '\t out = r_plfit(k,''hist'') works similar however one should note that\n'];
msg=[msg '\t in this mode r_plfit assumes by default that z=[1,2,...,W]\n'];
msg=[msg '\t The option ''urange'' has no effect in this mode and gets ignored if set\n'];
msg=[msg '\t Otherwise use out = r_plfit(k,''hist'',''range'',z) to set the event magnitudes z\n'];
msg=[msg '\t ''rangemin'' and ''rangemax'' options work in exactly the same way as before\n'];
msg=[msg '\n'];
msg=[msg 'Automatic low frequency cutoff \n'];
msg=[msg '\t By default r_plfit runs an iterative search for an optimal low frequency cutoff\n'];
msg=[msg '\t lf cutoff is set at index i where |Nz_i^lambda/N-Nmin| is minimal where Nmin=1\n'];
msg=[msg '\t by default and N the length of x=[x1,...,xN]; Nmin can be set using the\n'];
msg=[msg '\t ''Nmin'' option. If maxrange is smaller than the predicted lf cutoff then the\n'];
msg=[msg '\t cutoff has no effect. Note that in the out = r_plfit(k) mode the lf cutoff mechanism\n'];
msg=[msg '\t effectively acts as ahigh frequency cutoff with respect to the data x\n'];
msg=[msg '\t the option ''nolf''  switches of the lf cutoff \n'];
msg=[msg '\n'];
msg=[msg 'out = r_plfit(data,...,''plot'') displays the fit over the data\n'];
msg=[msg 'in double logarithmic coordinates (loglog plot)\n'];
msg=[msg '''fig'' behaves like ''plot'' but explicitly opens a new figure\n'];
msg=[msg '''exp_min'' can be used to specify the minimal search value exp_min (default =0)\n'];
msg=[msg 'for out.exponent. Similarly r_plfit(data,...,''exp_max'',expmax) sets the minimal\n'];
msg=[msg ' search value (default =5) to expmax\n'];
msg=[msg '''eps'' ... set absolute error eps (default eps=1e-10) for out.exponent \n'];
msg=[msg '\n'];
msg=[msg 'Other options to control the performance of the algorithm:\n'];
msg=[msg '''Ncut'' ... specifies the number of values alpha in the search range\n'];
msg=[msg 'of out.exponent. After m iterations the precision of out.exponent becomes\n'];
```

```
  msg=[msg '(expmax-expmin)/(Ncut/2)^m => m(eps)~2*(log(expmax-expmin)-log(eps))/Ncut\n'];
  msg=[msg '''Nimplicit'' sets the maximal number of implicit iteration for finding \n the exponent (default 80) of iterations\n'];
  msg=[msg 'searching implicitly for out.exponent. one should use Nimplicit>m(eps)\n'];
  msg=[msg '''Ntail'' sets the maximal number of iterations for the lf cutoff\n'];
  msg=[msg '''info'' if set will output info over the run stored in out.info at runtime!\n'];
  msg=[msg 'Bug reports to: rudolf.hanel@meduniwien.ac.at\n'];
  fprintf(1,msg); out=msg;
  return;
end; end;
%%%%%%%%%%%%%%%%%%%%%%%%%%%%%%%%%%%%%%%%%%%%%%%%%%%%%%%%%%%%
% Initialization & Default values
%%%%%%%%%%%%%%%%%%%%%%%%%%%%%%%%%%%%%%%%%%%%%%%%%%%%%%%%%%%%
N=length(z); % number of data items
xflg=0; cleanxflg=0; histflg=0; cdatflg=0; rangemin=0; rangemax=Inf;
Nrangeitems_max=1e6; eps=1e-10; issmall=1e-50; alpha_min=0; alpha_max=5;
lfcutoff=1; plotflg=0; newfigflg=0; testflg=1; Nmin=1; Ninc=50; Ntail=80;
Nimplicit=80; Nrep_cdat=80; rep_min=3; Amlthres=0.1; infoflg=0;
runinfo='start ...\n';

%%%%%%%%%%%%%%%%%%%%%%%%%%%%%%%%%%%%%%%%%%%%%%%%%%%%%%%%%%%%
% varargin
%%%%%%%%%%%%%%%%%%%%%%%%%%%%%%%%%%%%%%%%%%%%%%%%%%%%%%%%%%%%
id=0;
while id<length(varargin),
  id=id+1;
  if ischar(varargin{id}),
    switch varargin{id},
        case 'exp_min', alpha_min = varargin{id+1}; id=id+1;
        case 'exp_max', alpha_max = varargin{id+1}; id=id+1;
        case 'Ntail', Ntail = varargin{id+1}; id=id+1;
        case 'Nimplicit', Nimplicit = varargin{id+1}; id=id+1;
        case 'Nmin', Nmin = varargin{id+1}; id=id+1;
        case 'Ncut', Ninc = varargin{id+1}; id=id+1;
        case 'range', xrange = varargin{id+1}; xflg=1; id=id+1;
        case 'rangemin', rangemin = varargin{id+1}; id=id+1;
        case 'rangemax', rangemax = varargin{id+1}; id=id+1;
        case 'urange', xflg=2;
                                  % for cont dat we require the urange
        case 'cdat', cdatflg=1; lfcutoff=0; xflg=2;
        case 'cdat2', cdatflg=1; testflg=2; lfcutoff=0; xflg=2;
        case 'hist', histflg=1;
        case 'nolf', lfcutoff=0;
        case 'info', infoflg=1;
        case 'plot', plotflg=1;
        case 'fig', plotflg=1; newfigflg=1;
        case 'cleanrange',cleanxflg=1;
        case 'eps', eps = varargin{id+1}; id=id+1;
        otherwise, msg=['no such argument [' varargin{id} '] ->skip ...\n'];
            runinfo=[runinfo msg]; if infoflg==1, fprintf(1,msg); end;
    end
  end
end

msg=['handling arguments ...\n'];
runinfo=[runinfo msg]; if infoflg==1, fprintf(1,msg); end;

%%%%%%%%%%%%%%%%%%%%%%%%%%%%%%%%%%%%%%%%%%%%%%%%%%%%%%%%%%%%
% handle hist property, data filtering etc ...
%%%%%%%%%%%%%%%%%%%%%%%%%%%%%%%%%%%%%%%%%%%%%%%%%%%%%%%%%%%%
if histflg==1, %data is histogram data
```

```
    msg=['handling histogram input case ' num2str(xflg), ' ...\n'];
    runinfo=[runinfo msg]; if infoflg==1, fprintf(1,msg); end;
    k=z; zz=z;
    %... urange property is specified
    if xflg==2,
                    msg=['r_plfit: urange property has no effect together with hist input -> ', ...
                    'set default range case 0 ... \n'];
                    runinfo=[runinfo msg]; if infoflg==1, fprintf(1,msg); end; xflg=0;
    end;
    switch xflg,     % if input is histogram set range to...
        case 0, %... default range if range is not specified
            x=1:length(k); rangemin=max(rangemin,1); rangemax=min(rangemax,length(k));
        case 1, %... range property is specified
            if length(k)==length(xrange); x=xrange;
                rangemin=max(rangemin,min(x)); rangemax=min(rangemax,max(x));
            else
                fprintf(1,runinfo);
                error('r_plfit: range and histogram must have same length!!!');
            end;
        otherwise
            fprintf(1,runinfo);
            error('this should not happen 1 !!!');
    end;
    xidv=find(x>=rangemin & x<=rangemax);
    W=length(x); N=sum(k); xx=x(xidv);
    kk=k(xidv); WW=length(xx); NN=sum(kk);
else
%%%%%%%%%%%%%%%%%%%%%%%%%%%%%%%%%%%%%%%%%%%%%%%%%%%%%%%
% data=samples; clean data
%%%%%%%%%%%%%%%%%%%%%%%%%%%%%%%%%%%%%%%%%%%%%%%%%%%%%%%
    msg=['cleaning data input ... \n'];
    runinfo=[runinfo msg];
    if infoflg==1, fprintf(1,msg); end;
    idv=find(z>0); zz=z(idv); idv=find(abs(log(zz))<Inf);
    zz=zz(idv); idv=find(isnan(zz)==0); zz=zz(idv);
        %%%%%%%%%%%%%%%%%%%%%%%%%%%%%%%%%%%%%%%%%%%%%%%%%%%%%%%
    rangemin=max(rangemin,min(zz));
    rangemax=min(rangemax,max(zz));
    if rangemin>rangemax,
        fprintf(1,runinfo);
        error('r_plfit requires rangemin<rangemax!');
    end;
    zidv=find(zz>=rangemin & zz<=rangemax); zz=zz(zidv);
        %%%%%%%%%%%%%%%%%%%%%%%%%%%%%%%%%%%%%%%%%%%%%%%%%%%%%%%
    if (((max(zz)-min(zz))>Nrangeitems_max) & (xflg==0)),
        msg1=['Length of r_plfit default range rangemin:1:rangemax ' ...
                              num2str((max(zz)-min(zz))) 'is too large!\n'];
        msg2=['Maximum allowed: ' num2str(Nrangeitems_max) ...
                              '; => r_plfit tries the urange option!\n'];
        msg3=['Otherwise, try again by setting the range property manually with options:' ...
                    ' range, maxrange, minrange!\n'];
        runinfo=[runinfo msg1 msg2 msg3];
        if infoflg==1, fprintf(1,[msg1 msg2 msg3]); end; xflg=2;
    end;
    msg=['handling data input case ' num2str(xflg), ' ...\n'];
    runinfo=[runinfo msg];
    if infoflg==1, fprintf(1,msg); end;
    switch xflg,
```

```
                                    %... default range if range is not
                                        specified
        case 0,
            x=min(zz):max(zz);
            rangemin=max(rangemin,min(x));
            rangemax=min(rangemax,max(x));
                                    %... range property is specified
        case 1,
            x=xrange;
            rangemin=max(rangemin,min(x));
            rangemax=min(rangemax,max(x));
            % filter
            uz=unique(zz); uzon=zeros(size(uz));
            for uid=1:length(uz),
                                                            v=find(uz(uid)==x);
              if length(v)>0, uzon(uid)=1; end;
            end;
            uzoffidv=find(uzon==0);
            for uid=1:length(uzoffidv),
              v=find(uz(uzoffidv(uid))==zz);
              if length(v)>0, zz(v)=-1; end;
            end;
            v=find(zz>0); zz=zz(v);
                                    %... urange property is specified
        case 2,
            x=unique(zz);
            rangemin=max(rangemin,min(x));
            rangemax=min(rangemax,max(x));
        otherwise
            fprintf(1,runinfo);
            error('this should not happen 1 !!!');
    end;
%%%%%%%%%%%%%%%%%%%%%%%%%%%%%%%%%%%%%%%%%%%%%%%%%%%%%%%%%%%%
% sort data & filter sample-space
%%%%%%%%%%%%%%%%%%%%%%%%%%%%%%%%%%%%%%%%%%%%%%%%%%%%%%%%%%%%
    zz=sort(zz);
    if cleanxflg==1,
        msg=['cleaning sample space ...\n'];
        runinfo=[runinfo msg];
        if infoflg==1, fprintf(1,msg); end;
        uz=unique(zz); xon=zeros(size(x));
        for xid=1:length(uz),
            v=find(uz==x(xid));
            if length(v)>0, xon(xid)=1; end;
        end;
        x=x(find(xon==1));
    end;
%%%%%%%%%%%%%%%%%%%%%%%%%%%%%%%%%%%%%%%%%%%%%%%%%%%%%%%%%%%%
% compute histogram
%%%%%%%%%%%%%%%%%%%%%%%%%%%%%%%%%%%%%%%%%%%%%%%%%%%%%%%%%%%%
    msg=['computing histogram ...\n'];
    runinfo=[runinfo msg]; if infoflg==1, fprintf(1,msg); end;
    x=sort(x); W=length(x); maxx=max(x);
    while 1,
        xidv=find(x>=rangemin & x<=rangemax);
        if length(xidv)==0,
                                            rangemin=max(rangemin-1,issmall);
                                            rangemax=min(rangemax+5,maxx);
        else break; end;
    end;
    k=zeros(size(x)); [k,xdummy]=hist(zz,x); N=sum(k);
                xx=x(xidv); kk=k(xidv); WW=length(xx); NN=sum(kk);
end;
[a,b]=size(kk); if b==1, kk=kk'; end;
[a,b]=size(xx); if b==1, xx=xx'; end;
```

```
%%%%%%%%%%%%%%%%%%%%%%%%%%%%%%%%%%%%%%%%%%%%%%%%%%%%%%%%%%%
%%%%%%%%%%%%%%%%%%%%%%%%%%%%%%%%%%%%%%%%%%%%%%%%%%%%%%%%%%%
% Maximum likelihood estimate: find ML optimal alpha
%%%%%%%%%%%%%%%%%%%%%%%%%%%%%%%%%%%%%%%%%%%%%%%%%%%%%%%%%%%
%%%%%%%%%%%%%%%%%%%%%%%%%%%%%%%%%%%%%%%%%%%%%%%%%%%%%%%%%%%
xrangemax=max(xx); xrangemax2=xrangemax;
idrmax=WW; idrmax_1=0; idrmax_2=0; QQ=kk.*log(xx);
xmin=rangemin; xmax=rangemax; alphbest=0;

param.Nimplicit=Nimplicit; param.Ninc=Ninc;
param.alpha_max=alpha_max; param.alpha_min=alpha_min;
param.cdatflg=cdatflg; param.testflg=testflg;
param.infoflg=infoflg; param.issmall=issmall;

if lfcutoff==1,
%%%%%%%%%%%%%%%%%%%%%%%%%%%%
%% optimize low frequency cut-off for ML optimal alpha
    idrmax_1=WW;
    msg=['find exponent (lf cut-off is on) ...\n'];
    runinfo=[runinfo msg];
    if infoflg==1, fprintf(1,msg); end;
    for rep1=1:Ntail,
        xridv=find(xx<=xrangemax2); idrmax_2=idrmax_1;
        idrmax_1=idrmax; idrmax=max(xridv); xxx=xx(xridv);
        kkk=kk(xridv); WWW=length(xridv); NNN=sum(kkk); QQQ=QQ(xridv);
%%%%%%%%%%%%%%%%%%%%%%%%%%%%
%%% optimize alpha for Ninc alpha values
        loopmsg=['lf-loop: ', num2str(rep1),' '];
        [alphbest,runinfo]=h_implicit(NNN,xxx,QQQ,runinfo,loopmsg,alphbest,param);
        Aml=alphbest; yml=xx.^(-Aml); yml=yml/sum(yml);
        gugu=abs(NN*yml-Nmin); v=find(gugu==min(gugu));
        if Aml>Amlthres, idrmax=max(v); else idrmax=WW; end;
        if (idrmax==idrmax_1 | idrmax==idrmax_2) & rep1>=rep_min, break;
            end;
        idrmax=max(idrmax,1); xrangemax2=xx(idrmax);
    end;
else
    msg=['find exponent (lf cut-off is off) ...\n'];
    runinfo=[runinfo msg];
    if infoflg==1, fprintf(1,msg); end;
    [alphbest,runinfo]=h_implicit(NN,xx,QQ,runinfo,'',alphbest,param);
    Aml=alphbest; idrmax=WW;
end
msg=['preparing output ...\n'];
runinfo=[runinfo msg];
if infoflg==1, fprintf(1,msg); end;

xlfcutoff=1:idrmax;

% Quality of fit;
xxx=xx(xlfcutoff);
plf=xxx.^(-Aml); %Aml,
plf=plf/sum(plf);
p=xx.^(-Aml); %Aml,
p=p/sum(p);

ylf=kk(xlfcutoff)/sum(kk(xlfcutoff)); y=kk/sum(kk);
if plotflg==1,
    if newfigflg==1, figure; end;
    loglog(xxx,ylf,'r')
    hold on;
    loglog(xxx,plf),
    loglog(xxx(idrmax),plf(idrmax),'go'),
    pause(0.01);
end;
```

```
out.runinfo=runinfo;
out.N = NN; out.K=kk; out.fit.data=zz;
out.fit.Nsamples=N; out.fit.Nmin = Nmin;
out.fit.range=xx; out.fit.lf_cutoff_on=lfcutoff;
out.fit.alpha_min=alpha_min; out.fit.alpha_max=alpha_max;
out.fit.Ntail=Ntail; out.fit.Nimplicit=Nimplicit;
out.fit.Ninc=Ninc;

out.exponent = Aml;
out.KSall = max(abs(cumsum(y)-cumsum(p)));
out.KSrange = max(abs(cumsum(ylf)-cumsum(plf)));
out.xmax = max(xx); out.xmin = min(xx);
out.rangemax = rangemax; out.rangemin = rangemin;
out.lf_rangemax = max(xxx); out.lf_cutoffid = idrmax;

return;
%%%%%%%%%%%%%%%%%%%%%%%%%%%%%%%%%%%%%%%%%%%%%%%%%%%%%%%
%%%%%%%%%%%%%%%%%%%%%%%%%%%%%%%%%%%%%%%%%%%%%%%%%%%%%%%
% MAIN END
%%%%%%%%%%%%%%%%%%%%%%%%%%%%%%%%%%%%%%%%%%%%%%%%%%%%%%%
%%%%%%%%%%%%%%%%%%%%%%%%%%%%%%%%%%%%%%%%%%%%%%%%%%%%%%%

function [alphbest,runinfo]=h_implicit(NN,xx,QQ,runinfo,loopmsg,alphbest,param)

Nimplicit=param.Nimplicit; Ninc=param.Ninc;
alpha_max=param.alpha_max; alpha_min=param.alpha_min;
cdatflg=param.cdatflg; testflg=param.testflg;
infoflg=param.infoflg; issmall=param.issmall;
xmin=min(xx); xmax=max(xx);

dalph=(alpha_max-alpha_min)/Ninc;
alphav=alpha_min:dalph:alpha_max;
QQQ=sum(QQ);
for rep2=1:Nimplicit,
    msg1=['implicit: ', num2str(rep2)];
    msg2=[' accuracy: ' ,num2str(log(dalph/eps)/log(10))];
    msg3=[' alpha: ', num2str(alphbest),' ...\n'];
    runinfo=[runinfo loopmsg msg1 msg2 msg3];
    if infoflg==1, fprintf(1,[loopmsg msg1 msg2 msg3]); end;
    d=zeros(size(alphav));
    count=0;
    logxx=log(xx);
    for alph=alphav,
        count=count+1; px=xx.^(-alph); px=px/sum(px);
        if cdatflg==0
            FFF=NN*sum(px.*logxx);
        else
            if abs(alph-1)<issmall, if alph<1 alph=1-issmall; else alph=1+issmall; end; end;
            %%%%%%%%%%%%%%%%%%%%%%%%%%%%%%%%%%%%%%%%%%%%%%%
            switch testflg,
                case 1,
                % PRIMITIVE ESTIMATOR FOR xmax and xmin ....
                    dhi=0; dlo=0;
                    xmax2=xmax+dhi;
                    xmin2=xmin-dlo;
                    hxmax=xmax2.^(1-alph);
                    hxmin=xmin2.^(1-alph);
                    if hxmax>hxmin, h1=max(hxmax-hxmin,issmall);
                    else h1=-max(hxmin-hxmax,issmall);
                    end;
                    FFF=NN*((hxmax*log(xmax2)-hxmin*log(xmin2))/h1-1/(1-alph));
                case 2,
                % EXPERIMENTAL ESTIMATOR FOR xmax and xmin ....
                % REM: works quite badly!!!
```

```
                        lxx=length(xx);
                        hxhi=xx(3:lxx);
                        hxlo=xx(1:(lxx-2));
                        hxmid=xx(2:(lxx-1));
                        hf1=(hxhi-hxlo).*hxmid.^(1-alph);
                        hf2=hf1.*log(hxmid);
                        FFF=NN*sum(hf2)/sum(hf2);
                    otherwise
                        error('... this should never happen!');
                end;
            end;
            val=abs(QQQ-FFF);
            if isnan(val)==0; d(count)=val; else d(count)=Inf; end;
        end;
        if length(d)==0,
            msg=['dalpha too small -> break loop: ', dalph, ' !!!\n'];
            runinfo=[runinfo msg];
            if infoflg==1, fprintf(1,msg); end;
            break;
        end;
        v=find(d==min(d));
        alphbest=alphav(v(1));
        new_dalph=4*dalph/Ninc;
        alphav=(alphbest-2*dalph):new_dalph:(alphbest+2*dalph);
        if dalph<eps, break; end;
        dalph=new_dalph;
    end;
```

APPENDIX D2: r_plhistfit

```

%%%%%%%%%%%%%%%%%%%%%%%%%%%%%%%%%%%%%%%%%%%%%%%%%%%%%%%%
% Author: R Hanel
%%%%%%%%%%%%%%%%%%%%%%%%%%%%%%%%%%%%%%%%%%%%%%%%%%%%%%%%
function out = r_plhistfit(k,varargin)
%%%%%%%%%%%%%%%%%%%%%%%%%%%%%%%%%%%%%%%%%%%%%%%%%%%%%%%%
% latest modification on 12.07.2016
%%%%%%%%%%%%%%%%%%%%%%%%%%%%%%%%%%%%%%%%%%%%%%%%%%%%%%%%
% arg
%%%%%%%%%%%%%%%%%%%%%%%%%%%%%%%%%%%%%%%%%%%%%%%%%%%%%%%%
% k ... is the recorded data as a histogram
%       By default we assume a linear binning that
%       uses bins b_i=i+1/2 for bin k_i=|{x|b_i>x>b_{i-1}}|
%%%%%%%%%%%%%%%%%%%%%%%%%%%%%%%%%%%%%%%%%%%%%%%%%%%%%%%%
if length(k)==length('help'),
if strcmp(k,'help')==1,
    msg=['Using function out = r_plfit(data,varargin)\n'];
    msg=[msg 'r_plhistfit can be used to fit the exponent out.exponent of power laws\n'];
    msg=[msg 'where data is given as histogram k=[k_1, ... , k_W] \n'];
    msg=[msg 'The histogram is associated with bin margins b=[b_0, ... , b_W] \n'];
    msg=[msg 'such that each event x counted in the i''th bin fulfills b_i>x>b_{i-1}\n'];
    msg=[msg '\n'];
    msg=[msg 'Fitting binned histograms:\n'];
    msg=[msg '\t By default r_plhistfit(k) assumes that k is a histogram\n'];
    msg=[msg '\t over bins with margins b_i=i+1/2 \n'];
    msg=[msg '\t if other margins get used one can set them by using \n'];
    msg=[msg '\t out = r_plhistfit(x,''margins'',margins) ... where margins is a vector [b_0,....b_W] \n'];
    msg=[msg '\t To specify a fit range zmax <= z_i <= zmin (default zmin=min(x), zmax=max(x))\n'];
    msg=[msg '\t ''rangemin'' and ''rangemax'' options can be used to set the fit range\n'];
```

```
    msg=[msg '\n'];
    msg=[msg 'Automatic low frequency handling \n'];
    msg=[msg '\t By default r_plhistfit does not merge bins in such a way that each bin has at least Nmin elements  \n'];
    msg=[msg '\t the option ''lf''  switches on this lf handling \n'];
    msg=[msg '\n'];
    msg=[msg 'out = r_plfit(data,...,''plot'') displays the fit over the data\n'];
    msg=[msg 'in double logarithmic coordinates (loglog plot)\n'];
    msg=[msg '''fig'' behaves like ''plot'' but explicitly opens a new figure\n'];
    msg=[msg '''exp_min'' can be used to specify the minimal search value exp_min (default =0)\n'];
    msg=[msg 'for out.exponent. Similarly r_plfit(data,...,''exp_max'',expmax) sets the minimal\n'];
    msg=[msg ' search value (default =5) to expmax\n'];
    msg=[msg '''eps'' ... set absolute error eps (default eps=1e-5) for out.exponent \n'];
    msg=[msg '\n'];
    msg=[msg 'Other options to control the performance of the algorithm:\n'];
    msg=[msg '''Ncut'' ... specifies the number of values alpha in the search range\n'];
    msg=[msg 'of out.exponent. After m iterations the precision of out.exponent becomes\n'];
    msg=[msg '(exp_max-exp_min)/(Ncut/2)^m => m(eps)~2*(log(expmax-expmin)-log(eps))/Ncut\n'];
    msg=[msg '''Nimplicit'' sets the maximal number of implicit iteration for finding \nthe exponent (default 80) of iterations\n'];
    msg=[msg 'searching implicitly for out.exponent. one should use Nimplicit>m(eps)\n'];
    msg=[msg '''info'' if set will output info over the run stored in out.info at runtime!\n'];
    msg=[msg '''autocenter'' assumes that bins have an overall offset that can be optimized!\n'];
    msg=[msg '''eps2'' can be used to set the search accuracy of the ''autocenter'' option (default eps2=1e-7)!\n'];
    msg=[msg 'Bug reports to: rudolf.hanel@meduniwien.ac.at\n'];
    fprintf(1,msg);
    out=msg;
    return;
end;
end;
%%%%%%%%%%%%%%%%%%%%%%%%%%%%%%%%%%%%%%%%%%%%%%%%
% Initialization
%%%%%%%%%%%%%%%%%%%%%%%%%%%%%%%%%%%%%%%%%%%%%%%%
% number of data items
Nbin=length(k);
%%%%%%%%%%%%%%%%%%%%%%%%%%%%%%%%%%%%%%%%%%%%%%%%
% Default values

rangemin=0; rangemax=Inf; eps=1e-5; eps2=1e-7;
issmall=1e-10; smallaszero=1e-50;
alpha_min=0; alpha_max=5; xflg=0; lfflg=0;
plotflg=0;newfigflg=0; infoflg=0; centerflg=0;
centerfac=0; autocenterflg=0;
Nmin=1; Ncut=100; Nimplicit=500; Nautoc=50;
acenterf=0.999;

runinfo=['start ... \n'];

%%%%%%%%%%%%%%%%%%%%%%%%%%%%%%%%%%%%%%%%%%%%%%%%
% varargin
%%%%%%%%%%%%%%%%%%%%%%%%%%%%%%%%%%%%%%%%%%%%%%%%
id=0;
while id<length(varargin),
```

```
  id=id+1;
  if ischar(varargin{id}),
    switch varargin{id},
        case 'alpha_min', alpha_min = varargin{id+1}; id=id+1;
        case 'alpha_max', alpha_max = varargin{id+1}; id=id+1;
        case 'Nimplicit', Nimplicit = varargin{id+1}; id=id+1;
        case 'Nautoc', Nautoc = varargin{id+1}; id=id+1;
        case 'Nmin', Nmin = varargin{id+1}; id=id+1;
        case 'Ncut', Ncut = varargin{id+1}; id=id+1;
        case 'margins', margin = varargin{id+1}; xflg=1; id=id+1;
        case 'rangemin', rangemin = varargin{id+1}; id=id+1;
        case 'rangemax', rangemax = varargin{id+1}; id=id+1;
        case 'lf', lfflg = 1;
        case 'plot', plotflg=1;
        case 'plot2', plotflg=2;
        case 'fig', plotflg=1; newfigflg=1;
        case 'eps', eps = varargin{id+1}; id=id+1;
        case 'eps2', eps2 = varargin{id+1}; id=id+1;
        case 'autocenter', autocenterflg = 1;
        if centerflg==1,
                msg=['The option ''autocenter'' supersedes' ...
                ' the options ''center'' and ''centering''!!!\n'];
                runinfo=[runinfo msg];
                if infoflg==1, fprintf(1,msg); end;
                centerflg = 0;
        end;
        case 'acf', acenterf = varargin{id+1}; id=id+1;
        case 'center', % Ok
        if autocenterflg==1,
                msg=['The option ''autocenter'' supersedes' ...
                ' the options ''center'' and ''centering''!!!\n'];
                runinfo=[runinfo msg];
                if infoflg==1, fprintf(1,msg); end;
                centerflg = 0;
        else
                centerflg = 1;
        end;
        case 'centering', % Ok
        if autocenterflg==1,
                msg=['The option ''autocenter'' supersedes' ...
                ' the options ''center'' and ''centering''!!!\n'];
                runinfo=[runinfo msg];
                if infoflg==1, fprintf(1,msg); end;
                centerflg = 0; centerfac = 0;
        else
        centerflg = 1;
        centerfac = varargin{id+1};
        centerfac=min(max(centerfac,-1),1);
        end;
        id=id+1;
        case 'info',
        infoflg=1;
        otherwise,
        msg=['no such argument [' varargin{id} '] ->skip\n'];
        runinfo=[runinfo msg];
        if infoflg==1, fprintf(1,msg); end;
    end
  end
end
%%%%%%%%%%%%%%%%%%%%%%%%%%%%%%%%%%%%%%%%%%%%%%%%%%%%%%
% handle histogram, data filtering etc ...
%%%%%%%%%%%%%%%%%%%%%%%%%%%%%%%%%%%%%%%%%%%%%%%%%%%%%%
% data=histogram
%%%%%%%%%%%%%%%%%%%%%%%%%%%%%%%%%%%%%%%%%%%%%%%%%%%%%%
%     k=z;
msg=['handling histogram ...\n'];
```

```
runinfo=[runinfo msg];
if infoflg==1, fprintf(1,msg); end;

%if imput is histogram set range to...
switch xflg,
%...default binning if only histogram available
    case 0,
        msg=['setting default bins ...\n'];
        runinfo=[runinfo msg];
        if infoflg==1, fprintf(1,msg); end;
        margin=(1:(Nbin+1));
        if centerflg==1, margin=margin-1/2*(1+(1-issmall)*centerfac); end;
    %...binning if margins are available
    case 1,
        if length(margin)~=(Nbin+1),
            msg=['length(margin)=', num2str(length(margin)),' needst to equal length(k)+1=', ...
                                                              num2str(length(k)+1),'!!!'];
            error(msg);
        end;
        margin=sort(margin,'ascend');
    otherwise
        error('this should not happen 1 !!!');
end;
% remark here bin [margin(i),margin(i+1)] is associated with k(i+1).
rangemin=max(rangemin,margin(1));
rangemax=min(rangemax,max(margin)+issmall);
xidv=find(margin>=rangemin & margin<rangemax);
xmargin=margin(xidv); xNmarg=length(xmargin);
xNbin=xNmarg-1; kidv=xidv(2:xNmarg)-1; xk=k(kidv);
N=sum(k); W=length(k); NN=sum(xk); WW=length(xk);

if autocenterflg==1,
    msg=['handling autocenter option ...\n'];
    runinfo=[runinfo msg];
    if infoflg==1, fprintf(1,msg); end;
    acenterf=min(max(acenterf,0),1-issmall);
    dbfac=rangemin*acenterf;
    deltacv=2/Ncut;
    acv=(-1:deltacv:1)*dbfac;
    deltacv=deltacv*dbfac;
else
    Nautoc=1;
    deltacv=0;
    acv=[0];
end;
lacv=length(acv);

%%%%%%%%%%%%%%%%%%%%%%%%%%%%%%%%%%%%%%%%%%%%%%%%%%%%
% lf handling
%%%%%%%%%%%%%%%%%%%%%%%%%%%%%%%%%%%%%%%%%%%%%%%%%%%%
if lfflg==1,
    msg=['handling low frequency option ...\n'];
    runinfo=[runinfo msg];
    if infoflg==1, fprintf(1,msg); end;

    binsum=0;
    lfmarg=[margin(1)];
    lfk=[];
    mid=0;
    for id=1:WW,
        binsum=binsum+xk(id);
        if binsum>=Nmin,
            mid=id+1;
            lfmarg=[lfmarg xmargin(mid)];
```

```
                lfk=[lfk binsum];
                binsum=0;
            end;
        end
        % if the last bin margin is not xmarg(WW+1)
        lfW=length(lfmarg)-1;
        lfmarg(lfW+1)=xmargin(WW+1);
        lfk(lfW)=lfk(lfW)+binsum;
        xmargin=lfmarg; xk=lfk; WW=lfW;
    end;
    %%%%%%%%%%%%%%%%%%%%%%%%%%%%%%%%%%%%%%%%%%%%%%%%%%%%%%%%%%%%%
    % Maximum likelihood estimate & find optimal alpha
    %%%%%%%%%%%%%%%%%%%%%%%%%%%%%%%%%%%%%%%%%%%%%%%%%%%%%%%%%%%%%
    for rep1=1:Nautoc,
        xKSV=zeros(1,lacv);
        xAmlV=zeros(1,lacv);
        xcount=0;
        for dcent=acv,
            xcount=xcount+1; xxmargin=xmargin+dcent; idrmax=WW+1;
            xmax=max(xxmargin); xmin=min(xxmargin);
            dalph=(alpha_max-alpha_min)/Ncut;
            alphav=alpha_min:dalph:alpha_max;
            idrmax=length(xxmargin); p=zeros(1,WW); f=xk/NN;
            %%% optimize alpha for Ncut alpha values
            %xmargin0=xmargin;
            msg=['start optimizing exponent ...\n'];
            runinfo=[runinfo msg];
            if infoflg==1, fprintf(1,msg); end;
            %%%%%%%%%%%%%%%%%%%%%%%%%%%%%%%%%%%%%%%%%%%%%%%%%%%%%%%%%%%%%
            % Inner Iterations
            %%%%%%%%%%%%%%%%%%%%%%%%%%%%%%%%%%%%%%%%%%%%%%%%%%%%%%%%%%%%%
            for rep2=1:Nimplicit,
                d=zeros(size(alphav)); count=0;
                for alph=alphav,
                    count=count+1;
                    if abs(alph-1)<smallaszero,
                        hmarg=log(xxmargin);
                        hxmax=log(xmax);
                        hxmin=log(xmin);
                    else
                        hmarg=xxmargin.^(1-alph);
                        hxmax=xmax.^(1-alph);
                        hxmin=xmin.^(1-alph);
                    end;
                    p=hmarg(2:(WW+1))-hmarg(1:WW);
                    p=p/(hxmax-hxmin);
                    h1=hmarg.*log(xxmargin);
                    h2=h1(2:(WW+1))-h1(1:WW);
                    h3=hxmax*log(xmax)-hxmin*log(xmin);
                    p(find(p<smallaszero))=smallaszero;
                    p=p/sum(p);
                    m=h2/h3;
                    d(count)=abs(sum(f.*m./p)-1);
                end;
                if length(d)==0,
                    msg=['dalpha too small -> break loop: ', dalph, ' !!!\n'];
                    runinfo=[runinfo msg];
                    if infoflg==1, fprintf(1,msg); end;
                    break;
                end;
                v=find(d==min(d));
                alphbest=alphav(v(1));
                new_dalph=4*dalph/Ncut;
                alphav=(alphbest-2*dalph):new_dalph:(alphbest+2*dalph);
                if dalph<eps, break; end;
                dalph=new_dalph;
```

```
                msg1=['repetition:  ', num2str([rep1,rep2])];
                msg2=[' accuracy:  '
                    ,num2str(log([deltacv/eps2,dalph/eps])./log(10))];
                msg3=[' alpha:  ', num2str(alphbest),' ...\n'];
                msg=[msg1 msg2 msg3];
                runinfo=[runinfo msg];
                if infoflg==1, fprintf(1,msg); end;
            end;
%%%%%%%%%%%%%%%%%%%%%%%%%%%%%%%%%%%%%%%%%%
% End inner iterations
%%%%%%%%%%%%%%%%%%%%%%%%%%%%%%%%%%%%%%%%%%
            Aml=alphbest;
            if abs(Aml-1)<smallaszero,
                hmarg=log(xxmargin);
                hxmax=log(xmax);
                hxmin=log(xmin);
            else
                hmarg=xxmargin.^(1-Aml);
                hxmax=xmax.^(1-Aml);
                hxmin=xmin.^(1-Aml);
            end;
            pml=hmarg(2:(WW+1))-hmarg(1:WW);
            pml=pml/(hxmax-hxmin);
            %%%%%%%%%%%%%%%%%%%%%
            % Quality of fit;
            %%%%%%%%%%%%%%%%%%%%%
            fml=xk/sum(xk);
            KS = max(abs(cumsum(pml)-cumsum(fml)));
            xAmlV(xcount)=Aml;
            xKSV(xcount)=KS;
            %%%%%%%%%%%%%%%%%%%%%
            if plotflg>1,
                xdelta=diff(xxmargin);
                xcentroid=zeros(1,WW);
                xcentroid=(xxmargin(1:WW)+xxmargin(2:(WW+1)))/2;
                hold off;
                loglog(xcentroid,fml./xdelta,'r')
                hold on;
                loglog(xcentroid,pml./xdelta),
                pause(0.01);
            end;
                    %%%%%%%%%%%%%%%%%%%%%%%%%%%%%%%%%%%%%%%%%%
        end;
        v=min(find(xKSV==min(xKSV)));
        xid=v(1);
        Aml=xAmlV(xid);
        KSV=xKSV(xid);
        db=acv(xid);
        acvmin=db-2*deltacv;
        acvmax=db+2*deltacv;
        deltacv=4*deltacv/Ncut;
        acv=acvmin:deltacv:acvmax;
        lacv=length(acv);
        if deltacv<eps2, break; end;
end;
%%%%%%%%%%%%%%%%%%%%%%%%%%%%%%%%%%%%%%%%%%
% End outer iterations
%%%%%%%%%%%%%%%%%%%%%%%%%%%%%%%%%%%%%%%%%%

msg=['preparing output ...\n'];
runinfo=[runinfo msg];
if infoflg==1, fprintf(1,msg); end;

xxmargin=xmargin+db;
xmax=max(xxmargin);
xmin=min(xxmargin);
```

```
if abs(Aml-1)<smallaszero,
    hmarg=log(xxmargin);
    hxmax=log(xmax);
    hxmin=log(xmin);
else
    hmarg=xxmargin.^(1-Aml);
    hxmax=xmax.^(1-Aml);
    hxmin=xmin.^(1-Aml);
end;
pml=hmarg(2:(WW+1))-hmarg(1:WW);
pml=pml/(hxmax-hxmin);
%%%%%%%%%%%%%%%%%%%%
xcentroid=zeros(1,WW);
xcentroid=(xxmargin(1:WW)+xxmargin(2:(WW+1)))/2;
fml=xk/sum(xk);
%%%%%%%%%%%%%%%%%%%%
% plot
%%%%%%%%%%%%%%%%%%%%
xdelta=diff(xmargin); %/(max(xmargin)-min(xmargin));
hold off;
if plotflg>0,
    if newfigflg==1, figure; end;
    loglog(xcentroid,fml./xdelta,'r')
    hold on;
    loglog(xcentroid,pml./xdelta),
    pause(0.01);
end;
%%%%%%%%%%%%%%%%%%%%
% output
%%%%%%%%%%%%%%%%%%%%
out.runinfo=runinfo; out.exponent = Aml;
out.KS = KS; out.xmin = xmin; out.xmax = xmax;
out.pml = pml; out.f = fml; out.centers=xcentroid;
out.xmargin=xmargin; out.offset=db; out.keff=xk;
out.fit.N = N; out.fit.Neff= NN;
out.fit.alpha_min=alpha_min; out.fit.alpha_max=alpha_max;
out.fit.Nmin = Nmin; out.fit.Nimplicit=Nimplicit;
out.fit.Ncut=Ncut; out.fit.lfmode = lfflg;
out.fit.margin=margin; out.fit.k=k;
out.fit.delta=dalph;

return;
```

APPENDIX D3: r_plfit_calibrate

```
%%%%%%%%%%%%%%%%%%%%%%%%%%%%%%%%%%%%%%%%%%%%%%%%%%%
% Author: R Hanel
%%%%%%%%%%%%%%%%%%%%%%%%%%%%%%%%%%%%%%%%%%%%%%%%%%%
function out = r_plfit_calibrate(alpha,W, Nsamples, Nrep)
%%%%%%%%%%%%%%%%%%%%%%%%%%%%%%%%%%%%%%%%%%%%%%%%%%%
% USE FOR SAMPLING CALIBRATION CURVES FOR
% the r_plfit KS statistics.
% r_plfit_calibrate samples from a power-law with exponent
% alpha Nsamples (can be a vector) samples then uses r_plfit to predict alpha
% and measures the corresponding KS values. This is repeated
% for Nrep times. By sorting the KS vector of length Nrep
% in a descending order one gets a curve KS(id) over p=id/Nrep, which
% represents the p-value of the KS value KS(id) of the r_plfit estimate
% This means that for KS(id) there exists a chance p=id/Nrep that we
% might sample a KS value more extreme than KS(id). This allows us to
% determine the critical KS value KS(id) for which we may expect
% to reject only a fraction p=id/Nrep of samples of size Nsamples
% that actually have been drawn from a power-law with exponent alpha
%%%%%%%%%%%%%%%%%%%%%%%%%%%%%%%%%%
% out ... is a struct that returns a summary of the input
```

```
% out.alpha=alpha;
% out.W=W;
% out.Nsample=Nsamples;
% out.Nrep=Nrep;
% out.KSS=KSS;        ... the Nrep x length(Nsamples) matrix of KS statistics values
% out.LAM=LAM;        ... the Nrep x length(Nsamples) matrix of reconst. exponents
% out.KSSsorted=KSSsorted; ... the sorted matrix of KS statistics matrix
% out.LAMsorted=LAMsorted;  ... the sorted matrix of exponents (ordered in the same way as KS)
% out.Pval=Pvalv;    ... the vector p=(1:Nrep)/Nrep
%
%%%%%%%%%%%%%%%%%%%%%%%%%%%%%%%%%%%%%%%%%%%%%%%%%%%%%%%%%%%%
% define distribution to sample from
p=(1:W).^(-alpha); p=p/sum(p);
L=length(Nsamples);
Nsm=max(Nsamples);
%%%%%%%%%%%%%%%%%%%%%%%%%%%%%%%%%%%%%%%%%%%%%%%%%%%%%%%%%%%%
LAM=zeros(Nrep,L);  % the expected exponent
KSS=zeros(Nrep,L);  % the KS statistics variable
% produce Nrep data sets
for id=1:Nrep,
    fprintf(1,['---------- togo=', num2str(Nrep-id), '----------------\n']);
    data=r_randi(p,'p','Nsamples',Nsm);
    % look at sample-sizes N
    for id2=1:L;
        N=Nsamples(id2);
        [pdf1,dummy]=hist(data(1:N),1:W);
        pdf1=pdf1/sum(pdf1);
        % make the ML estimate of the exponent
        x=r_plfit(data(1:N),'noks');
        % and compute the estimated power-law distribution
        pdf2=(1:W).^x.exponent;
        pdf2=pdf2/sum(pdf2);
        % compute the KS value statistics
        KSS(id,id2) = max(abs(cumsum(pdf1)-cumsum(pdf2)));
        LAM(id,id2) = x.exponent;
    end;
end
KSSsorted=zeros(size(KSS));
LAMsorted=zeros(size(LAM));
Pvalv=(1:Nrep)/Nrep;
KSScrit=zeros(Nrep,L);
% compute the p-value landscape
for id2=1:L,
    [KSSv,I]=sort(KSS(:,id2),'descend');
    LAMv=LAM(I,id2);
    KSSsorted(:,id2)=KSSv;
    LAMsorted(:,id2)=LAMv;
end

% set the output
out.alpha=alpha;
out.W=W;
out.Nrep=Nrep;
out.KSS=KSS;
out.LAM=LAM;
out.KSSsorted=KSSsorted;
out.LAMsorted=LAMsorted;
out.Pval=Pvalv;
out.Nsample=Nsamples;
```

APPENDIX D4: r_plfit_calib_eval

```
%%%%%%%%%%%%%%%%%%%%%%%%%%%%%%%%%%%%%%%%%%%%%%%%%%%%
% Author: R Hanel
%%%%%%%%%%%%%%%%%%%%%%%%%%%%%%%%%%%%%%%%%%%%%%%%%%%%
function out = r_plfit_calib_eval(in,confidence,samplesize,plotflg)
%%%%%%%%%%%%%%%%%%%%%%%%%%%%%%%%%%%%%%%%%%%%%%%%%%%%
% finds the critical KS value from the confidence p-value confidence
% for the samples-size samplesize from in=r_plfit_calibrate(...) and returns
% out.confidence
% out.samplesize
% out.Nsample ... vector of sample sizes (r_plfit_calibrate)
% out.Pval ... (r_plfit_calibrate)
% out.KS ... selected (by confidence) and interpolated KS vector of length(out.Nsamples)
% out.KScrit ... selected (by confidence and smplesize) and interpolated critical KS value
%%%%%%%%%%%%%%%%%%%%%%%%%%%%%%%%%%%%%%
plotflg=round(plotflg);
if plotflg>2, plotflg=2; end;
if plotflg<0, plotflg=0; end;
%plotflg=2; % plot a calibration curve 1D + 2D
%plotflg=1; % plot a calibration curve 1D
%plotflg=0; % no plots
%%%%%%%%%%%%%%%%%%%%%%%%%%%%%%%%%%%%%%
L=length(in.Nsample);
Nrep=in.Nrep;

out.confidence=confidence;
out.samplesize=samplesize;
out.Nsample=in.Nsample;
out.Pval=in.Pval;
v=find(abs(in.Pval-confidence)==min(abs(in.Pval-confidence)));
cid=min(v);
if in.Pval(cid)<confidence,
  if cid>1,
    a=(in.Pval(cid)-confidence)/(in.Pval(cid)-in.Pval(cid-1));
    b=(confidence-in.Pval(cid-1))/(in.Pval(cid)-in.Pval(cid-1));
    out.KS=a*in.KSsorted(cid,:)+b*in.KSsorted(cid-1,:);
  else
    out.KS=in.KSsorted(cid,:);
  end;
else
  if cid<length(in.Pval),
    a=(in.Pval(cid+1)-confidence)/(in.Pval(cid+1)-in.Pval(cid));
    b=(confidence-in.Pval(cid))/(in.Pval(cid+1)-in.Pval(cid));
    out.KS=a*in.KSsorted(cid+1,:)+b*in.KSsorted(cid,:);
  else
    out.KS=in.KSsorted(cid,:);
  end;
end;
v=find(abs(in.Nsample-samplesize)==min(abs(in.Nsample-samplesize)));
sid=min(v);
if in.Nsample(sid)>samplesize,
  if sid>1,
    a=(in.Nsample(sid)-samplesize)/(in.Nsample(sid)-in.Nsample(sid-1));
    b=(samplesize-in.Nsample(sid-1))/(in.Nsample(sid)-in.Nsample(sid-1));
    out.KScrit=a*out.KS(sid)+b*out.KS(sid-1);
  else
    out.KScrit=out.KS(sid);
  end;
else
  if sid<length(in.Nsample),
    a=(in.Nsample(sid+1)-samplesize)/(in.Nsample(sid+1)-in.Nsample(sid));
    b=(samplesize-in.Nsample(sid))/(in.Nsample(sid+1)-in.Nsample(sid));
    out.KScrit=a*out.KS(sid+1)+b*out.KS(sid);
  else
```

```
        out.KScrit=out.KS(sid);
    end;
end;

if plotflg==1,
        figure;
        L=length(out.KS);
        plot(out.Nsample,out.KS);
        xlabel('sample size');
        ylabel('KS');
end;
if plotflg==2,
        figure;
        subplot(2,2,1);
            mesh(in.Nsample,in.Pval,in.LAMsorted);
            xlabel('sample size');
            ylabel('confid.');
            zlabel('\lambda');
        subplot(2,2,2);
            mesh(in.Nsample,in.Pval,in.KSSsorted);
            xlabel('sample size');
            ylabel('confid.');
            zlabel('KS');
        subplot(2,2,3);
            for id=1:L
                gval=id/L;
                plot(in.Pval,in.KSSsorted(:,id),'Color',[1,gval,1-gval]); hold on;
            end;
            hold off;
            xlabel('confid.');
            ylabel('KS');
        subplot(2,2,4);
            for id=1:Nrep
                gval=id/Nrep;
                plot(in.Nsample,in.KSSsorted(id,:),'Color',[1,1-gval,gval]); hold on;
            end;
            hold off;
            xlabel('sample size');
            ylabel('KS');
end;
return;
```

APPENDIX D5: r_randi

```
%%%%%%%%%%%%%%%%%%%%%%%%%%%%%%%%%%%%%%%%%%%%%%%%%
% Author: R Hanel
%%%%%%%%%%%%%%%%%%%%%%%%%%%%%%%%%%%%%%%%%%%%%%%%%
function n=r_randi(X,varargin),
%%%%%%%%%%%%%%%%%%%%%%%%%%%%%%%%%%%%%%%%%%%%%%%%%
% varargin:
% a) n=r_randi(X)
%   X ... can be a vector of numbers > 0
%   n ... returns a vector of randomnumbers with 1<=n(id)<=X(id)
% b) n=r_randi(X,'Nsamples',Nsamples)
%   X ... is a number > 0
%   Nsamples ... is a number > 0
%   n ... returns a vector of lenghth Nsamples with randomnumbers 1<=n(id)<=X
% c) n=r_randi(X,'p')
%   X ... is a real vector > 0 representing a distribution function
%       X gets normalized by r_randi to X/sum(X)
%   n ... returns a number drawn randomly from id=1:length(X)
%       with probability X(id)/sum(X)of;
%       if the 'Nsamples' option is used n is a vector of length(X)
```

```
% c) n=r_randi(X,'cp')
%   X ... is a real monotonically increasing vector > 0 representing a cummulative
%        distribution function; X gets normalized by r_randi to X/X(length(X))
%   n ... returns a number drawn randomly from the distribution
%        characterized by the cummulative distribution
%%%%%%%%%%%%%%%%%%%%%%%%%%%%%%%%%%%%%%%%%%%%%%%%%%%%
id=0;
isdist=0;
Nsamples=1;
while id<length(varargin),
  id=id+1;
  if ischar(varargin{id}),
    switch varargin{id},
        case 'Nsamples',
            % X is distribution
            Nsamples = varargin{id+1};
            id=id+1;
        case 'p',
            % X is distribution
            isdist = 1;
        case 'cp',
            % X is cummulative distribution
            isdist = 2;
        otherwise,
            fprintf(1,['no such argument [' varargin{id} '] ->skip\n']);
    end
  end
end
%%%%%%%%%%%%%%%%%%%%%%%%%%%%%%%%%%%%%%%%%%%%%%%%%%%%
n=zeros(1,Nsamples);
if isdist==0,
    W=max(round(X),1);
    if length(X)>1,
        Nsamples=length(X);
        n=zeros(1,Nsamples);
        n=min(1+floor(rand(size(W)).*W),W);
    else
        for id=1:Nsamples,
            n(id)=min(1+floor(rand(size(W))*W),W);
        end;
    end
elseif isdist==2
    if X(length(X))~=1, X=X/X(length(X)); end;
    for id=1:Nsamples,
        v=find(rand<=X);
        n(id)=min(v);
    end;
else
    p=X/sum(X);
    csp=cumsum(p);
    for id=1:Nsamples,
%          if mod(id,1000)==0, Nsamples-id, pause(0.1); end;
        v=find(rand<=csp);
        n(id)=min(v);
    end;
end;

return;
```
